# A systematic review and meta-analysis of cold exposure and cardiovascular disease outcomes

**DOI:** 10.3389/fcvm.2023.1084611

**Published:** 2023-03-27

**Authors:** Jie-Fu Fan, Yu-Chen Xiao, Yi-Fei Feng, Lu-Yu Niu, Xing Tan, Jia-Cen Sun, Yue-Qi Leng, Wan-Yang Li, Wei-Zhong Wang, Yang-Kai Wang

**Affiliations:** Department of Marine Biomedicine and Polar Medicine, Naval Medical Center of PLA, Naval Medical University (Second Military Medical University), Shanghai, China

**Keywords:** low temperature, cold spell, cardiovascular disease, meta-analysis, climate

## Abstract

**Background:**

Cold exposure has been considered an essential risk factor for the global disease burden, while its role in cardiovascular diseases is still underappreciated. The increase in frequency and duration of extreme cold weather events like cold spells makes it an urgent task to evaluate the effects of ambient cold on different types of cardiovascular disease and to understand the factors contributing to the population's vulnerability.

**Methods:**

In the present systematic review and meta-analysis, we searched PubMed, Scopus, and Cochrane. We included original research that explored the association between cold exposure (low temperature and cold spell) and cardiovascular disease outcomes (mortality and morbidity). We did a random-effects meta-analysis to pool the relative risk (RR) of the association between a 1°C decrease in temperature or cold spells and cardiovascular disease outcomes.

**Results:**

In total, we included 159 studies in the meta-analysis. As a result, every 1°C decrease in temperature increased cardiovascular disease-related mortality by 1.6% (RR 1.016; [95% CI 1.015–1.018]) and morbidity by 1.2% (RR 1.012; [95% CI 1.010–1.014]). The most pronounced effects of low temperatures were observed in the mortality of coronary heart disease (RR 1.015; [95% CI 1.011–1.019]) and the morbidity of aortic aneurysm and dissection (RR 1.026; [95% CI 1.021–1.031]), while the effects were not significant in hypertensive disease outcomes. Notably, we identified climate zone, country income level and age as crucial influential factors in the impact of ambient cold exposure on cardiovascular disease. Moreover, the impact of cold spells on cardiovascular disease outcomes is significant, which increased mortality by 32.4% (RR 1.324; [95% CI 1.2341.421]) and morbidity by 13.8% (RR 1.138; [95% CI 1.015–1.276]).

**Conclusion:**

Cold exposure could be a critical risk factor for cardiovascular diseases, and the cold effect varies between disease types and climate zones.

**Systematic Review Registration:**

https://www.crd.york.ac.uk/PROSPERO, identifier: CRD42022347247.

## 1. Introduction

Climate change has a significant impact on human health and has become a global health concern (1–3). *Global disease burden 2019* reported that non-optimal temperatures accounted for 1.01 million deaths in males and 0.946 million in females (1). Despite the long-term warming trends, there is an increase in the number, frequency, and duration of extreme weather events such as cold spells, which makes cold exposure a more significant threat (4, 5). It has been reported that for every 1°C temperature decrease below the reference point, the rate of non-accidental mortality increases by 4% (6). Therefore, it is crucial to clarify the impact of cold exposure on human health outcomes.

Cardiovascular diseases (CVDs) are the leading cause of disease burden, accounting for nearly one-third of total deaths worldwide (1). In many countries, CVD mortality is higher in winter than in summer (7, 8). As reported, sudden exposure to low temperatures could disturb cardiovascular activity (9, 10). Cold exposure induces an increase in blood pressure and changes in blood components, which could induce disease conditions such as hypertension, myocardial infarction (MI), and atherosclerosis (7, 11, 12). This evidence suggests that cold exposure might be an essential risk factor for cardiovascular diseases and increase the health burden. Considering the increasing intensity and frequency of cold surges and cold spells (4), it is vital to demonstrate the impact of cold exposure on cardiovascular diseases.

Previous studies have reported a positive association between cold exposure and cardiovascular mortality and morbidity (6, 11, 13, 14). However, the extent of cold impact on cardiovascular health remains disputable. Specifically, Ren et al. reported a 14.3% increase in cardiovascular mortality followed by every 1°C decrease in temperature (15), while Bai et al. found only a 1.1% increase (16). The wide variation between studies hinders a proper understanding of cold impact. More importantly, the influential factors that cause variations are worth investigating. Previous meta-analyses mainly focused on cold impact on all-cause mortality, in which cardiovascular disease was discussed only as a subgroup. Hence, there is currently no study that systematically analyzes cold impact on different kinds of cardiovascular disease, let alone discusses the influential factors of cold impact such as climate zones. A review that focuses on cold impact on cardiovascular disease is crucial to provide more specific and detailed information on matters such as cold impact on different kinds of cardiovascular diseases, the vulnerabilities of the population, and influential factors.

Therefore, we conducted a wide-ranging search and analysis of the available epidemiological evidence concerning the effects of cold exposure (low temperatures and cold spells) on cardiovascular disease outcomes. We carried out an elaborate stratification on the included literature, examining cold impact on different kinds of diseases and exploring the susceptibility of the population to cardiovascular disease outcomes resulting from cold exposure.

## 2. Methods

We followed the preferred reporting items for systematic reviews and meta-analysis (PRISMA) guidelines to plan and conduct this review (17) (Supplementary Table S1), and the study protocol was registered with PROSPERO (CRD42022347247).

### 2.1. Literature search and selection criteria

We searched the databases of PubMed, Scopus, and Cochrane. Keywords such as “temperature”, “weather”, “climate change”, or “cold” were used for exposures. As for health outcomes, we used “cardiovascular disease”, “heart disease”, “vascular disease”, “cerebrovascular disease”, “hypertensive disease”, “myocardial infarction”, “stroke”, “heart failure”, “arrhythmia”, “cardiac arrest”, “rheumatic heart disease”, “thrombotic disease”, “pulmonary heart disease”, and “aortic aneurysm and dissection”. Peer-reviewed studies published in English before February 6, 2023, were identified. Reference lists of all selected articles were independently screened to identify additional studies left out in the initial search. These processes were developed by two investigators (JF and YC), and any differences in investigators’ decisions were discussed. The complete search strategy used for each database is outlined in Supplementary Table S2.

### 2.2. Eligibility criteria

**Figure 1:**
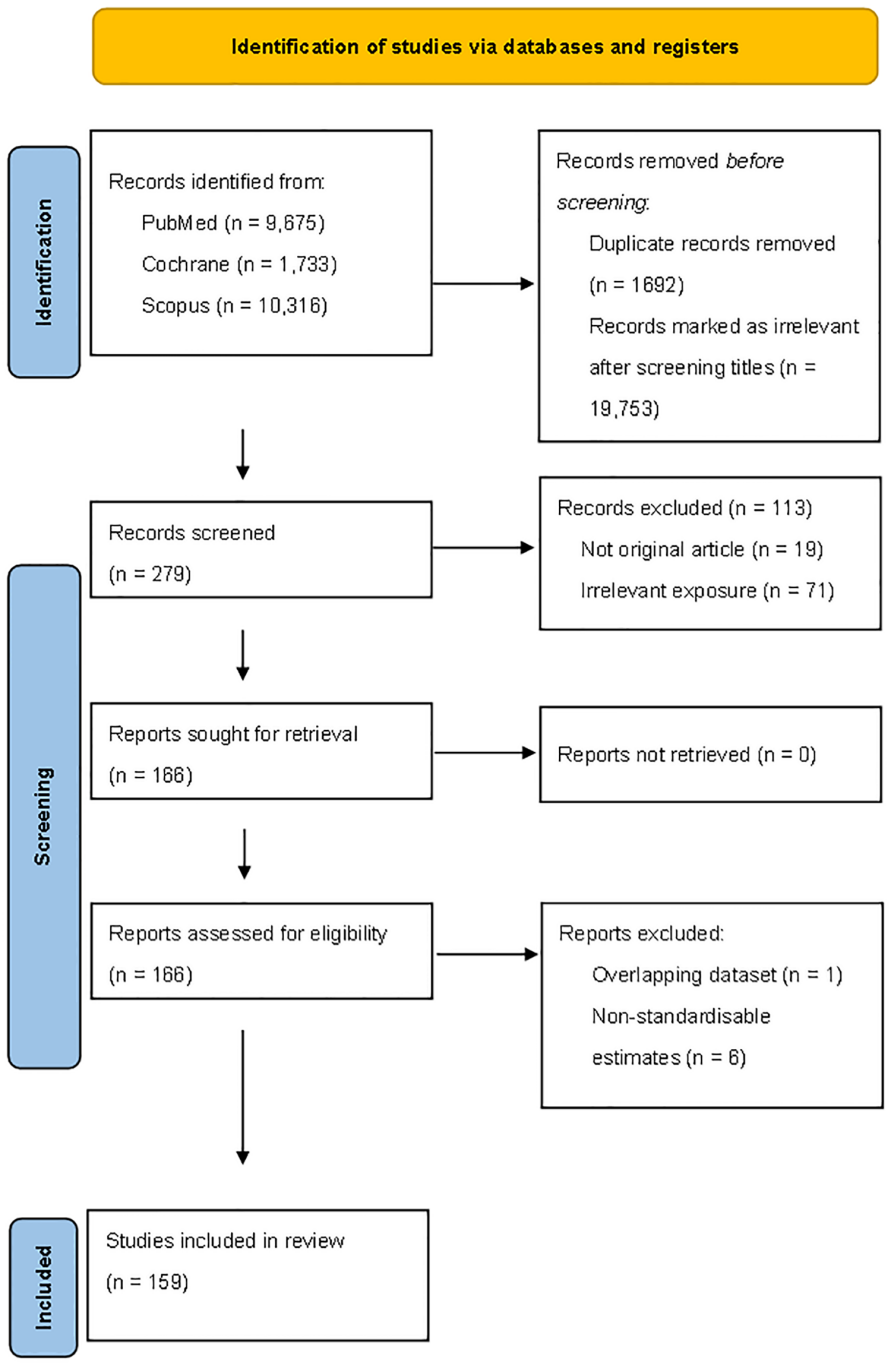
Flowchart of the assessment of eligible studies.

In the literature search, we included studies that met the following selection criteria: (1) original, peer-reviewed articles with an independent study population; (2) articles that included information on the relationship between cold exposure and cardiovascular-related mortality (death) and morbidity (hospitalization, emergency room visit, ambulance call-out, and out-of-hospital cardiac arrest); and (3) articles categorized as a time-series study or case-crossover study. For this review and meta-analysis, articles were excluded if they reported percentiles for exposure assessment or seasonal effects rather than specific temperatures. Figure 1 presents a flow diagram of the study selection process.

### 2.3. Data extraction

An Excel data extraction form was created to record study information on the study period, study population, exposure, outcome, and results on the effects of cold (Table 1). The summary estimates were obtained from the published tables and figures through textual descriptions and Supplementary Material. When information from the figures was imprecise or detailed data seemed available but not provided in the article, we contacted the authors to request further data. When both crude and adjusted estimates were reported, we used the adjusted estimates (18). If multiple studies were using the same data and were conducted by the same research group, we considered the results for the most recent publication. If different research groups conducted the studies, we included all of them in the pooled analysis.

**Table 1:** Characteristics of included studies.

ID	Author	Year	Location	Study period	Study design	Exposure	Mean value (°C), range	Mean value (°C)	Season	Adjusted for air pollution	Mortality/morbidity	Outcome (ICD)	Ages	Climate zones	Climate zones	Income group
1	Ren et al.	2006	Brisbane, Australia	1996–2001	TS	Mean	15.42 (1.2 to 26)	15.42	Annual	PM10, O3	Both	CVD (I00–I99)	All ages	Cfa	C-subtropical	H
2	Ferreira et al.	2019	Five cities, Brazil	1996–2013	TS	Mean	20.5 to 26.5 (4.9 to 31.9)	20.5	Annual	NA	Mortality	ACS (I21–I22)	All ages	Af	A-tropical	UM
3	Analitis et al.	2018	Nine cities, Europe	2004–2010	TS	Tapp	18.4 to 30 (NA to NA)	23	Cold (Oct–Mar)	PM10, O3, NO2	Mortality	CVD (I00–I99)	All ages	Csb	C-mediterranean	H
4	Hashizume et al.	2009	Matlab, Bangladesh	1994–2002	TS	Mean	NA (NA to NA)	NA	Annual	NA	Mortality	CVD (I00–I99)	All ages	Aw	A-tropical	LM
5	Huang et al.	2014	Changsha, China	2008–2011	TS	Cold spell	NA (−5.3 to 40.7)	NA	Annual	PM10, NO2, SO2	Mortality	CVD (I00–I99)	All ages	Cfa	C-subtropical	UM
6	Lin et al.	2013	Four metropolitans, Taiwan	1994–2007	TS	Mean	24.2 (8.1 to 33)	24.2	Annual	PM10, O3, NO2	Mortality	CVD (I00–I99)	All ages	Cfa	C-subtropical	H
7	Son et al.	2016	São Paulo, Brazil	1996–2010	TS	Mean	20.1 (7.5 to 28.7)	20.1	Annual	PM2.5, PM10, O3	Mortality	CVD (I00–I99)	All ages	Cfa	C-subtropical	UM
8	Xiong et al.	2017	Shanghai, China	2011–2013	TS	Mean	17.4 (−2 to 36)	17.4	Annual	PM10, NO2, SO2	Mortality	CVD (I00–I99)	All ages	Cfa	C-subtropical	UM
9	Ikefuti et al.	2018	São Paulo, Brazil	2002–2011	TS	Mean	19.5 (8.4 to 27.6)	19.5	Annual	PM10, O3, NO2,SO2	Mortality	Stroke (I60–I69)	All ages	Cfa	C-subtropical	UM
10	Gouveia et al.	2003	São Paulo, Brazil	1991–1994	TS	Mean	19.3 (7 to 26.3)	19.3	Annual	PM10, O3, NO2, CO, SO2	Mortality	CVD (I00–I99)	All ages	Cfa	C-subtropical	UM
11	Liu et al.	2020	Hong Kong, China	2007–2015	TS	Mean	23.5 (8.4 to 32.4)	23.5	Annual	PM2.5, O3, NO2, SO2	Mortality	CVD (I00–I99)	All ages	Cfa	C-subtropical	H
12	Guo et al.	2011	Tianjin, China	2005–2007	CC	Mean	13 (−7 to 29)	13	Annual	PM10, NO2, SO2	Mortality	CVD (I00–I99)	All ages	Dwa	D-continental	UM
13	Zhang et al.	2014	Five cities, China	2004–2008	TS	Mean	17.12 (−10.5 to 34.2)	17.12	Annual	PM10, NO2	Mortality	Stroke (I60–I69)	All ages	Dwa	D-continental	UM
14	Romani et al.	2020	Two cities, Spain	2005–2017	TS	Min	NA (−2.8 to 40.8)	NA	Annual	NA	Mortality	CVD (I00–I99)	All ages	Csb	C-mediterranean	H
15	Dai et al.	2015	Shanghai, China	2006–2011	TS	Mean	18 (−2 to 34)	18	Annual	PM2.5, PM10, O3, NO2, CO, SO2	Mortality	CHD (I20–I25)	All ages	Cfa	C-subtropical	UM
16	Silveira et al.	2019	27 cities, Brazil	2000–2015	TS	Mean	18.9 to 28.9 (3.8 to 36)	23.9	Annual	NA	Mortality	CVD (I00–I99)	All ages	Multi	Multi	UM
17	Guo et al.	2012	Chiang Mai, Thailand	1999–2008	TS	Mean	26.2 (13.3 to 33.5)	26.2	Annual	PM10, O3	Mortality	CVD (I00–I99)	All ages	Aw	A-tropical	UM
18	Seposo et al.	2015	Manila, Philippines	2006–2010	TS	Mean	28.8 (23.5 to 33.3)	18.8	Annual	NA	Mortality	CVD (I00–I99)	All ages	Aw	A-tropical	LM
19	Zhang et al.	2016	Wuhan, China	2003–2010	TS	Mean	17.9 (−2.7 to 35.8)	17.9	Annual	PM10, NO2, SO2	Mortality	CVD (I00–I99)	All ages	Cfa	C-subtropical	UM
20	Yang et al.	2012	Guangzhou, China	2003–2007	CC	Mean	23 (2.1 to 34.2)	23	Annual	PM10, NO2, SO2	Mortality	CVD (I00–I99)	All ages	Cfa	C-subtropical	UM
21	Yu et al.	2011	Brisbane, Australia	1996–2004	TS	Mean	20.1 (9.8 to 31.9)	20.1	Summer	PM10, O3, NO2	Mortality	CVD (I00–I99)	All ages	Cfa	C-subtropical	H
22	Yu et al.	2011	Brisbane, Australia	1996–2004	TS	Mean	20.1 (15.4 to 25.2)	20.1	Annual	PM10, O3, NO2	Mortality	CVD (I00–I99)	All ages	Cfa	C-subtropical	H
23	Kwon et al.	2015	South Korea	2004–2012	TS	Min	24.19 (10.2 to 32.7)	24.19	Cold (Dec–Feb)	PM10, O3, NO2, CO, SO2	Mortality	CVD (I00–I99)	All ages	Multi	Multi	H
24	Ma et al.	2020	Jiangsu, China	2015–2017	TS	Mean	13.9 (−11.5 to 30.6)	13.9	Annual	PM2.5, O3, NO2, CO, SO2	Both	CVD (I00–I99)	All ages	Cfa	C-subtropical	UM
25	Ballester et al.	1997	Valencia, Spain	1991–1993	TS	Mean	22 (NA to NA)	22	Cold (Nov–Apr)	SO2	Mortality	CVD (I00–I99)	All ages	BSk	B-dry	H
26	Silveira et al.	2021	Rio de Janeiro, Brazil	2001–2018	CC	Mean	24.7 (15.5 to 35)	24.7	Annual	NA	Mortality	CVD (I00–I99)	All ages	Aw	A-tropical	UM
27	Zhai et al.	2022	Qingdao, China	2009–2017	TS	Mean	14.5 (NA to NA)	14.5	Annual	NA	Mortality	CVD (I00–I99)	All ages	Cwa	C-subtropical	UM
28	Ma et al.	2014	17 cities, China	1996–2008	TS	Mean	15.3 (−23.7 to 36.4)	15.3	Annual	PM10, NO2, SO2	Mortality	CVD (I00–I99)	All ages	Multi	Multi	UM
29	O'Neill et al.	2005	Two cities, Mexico	1996–1998	TS	Tapp	19.9 (−2.7 to 42.1)	19.9	Annual	PM10, O3	Mortality	CVD (I00–I99)	All ages	Cwb	C-oceanic	UM
30	Yi and Chan	2015	Hong Kong, China	2002–2011	TS	Mean	23.4 (8.2 to 31.8)	23.4	Annual	PM10, NO2, SO2	Mortality	CVD (I00–I99)	All ages	Cfa	C-subtropical	H
31	Xing et al.	2020	Beijing, China	2006–2011	TS	Mean	12.56 (−14.1 to 33)	12.56	Annual	PM2.5, O3, NO2, SO2	Mortality	CVD (I00–I99)	All ages	Dwa	D-continental	UM
32	Sharovsky et al.	2004	São Paulo, Brazil	1996–1998	TS	Mean	19.3 (8.8 to 28.3)	19.3	Annual	PM10, NO2, CO	Mortality	ACS (I21–I22)	All ages	Cfa	C-subtropical	UM
33	Achebak et al.	2018	47 cities, Spain	1980–2015	TS	Mean	NA (NA to NA)	NA	Summer months	NA	Mortality	CVD (I00–I99)	All ages	Multi	Multi	H
34	Lin et al.	2020	Taiwan, China	2000–2014	TS	Mean	23.3 (9.5 to 31.1)	23.3	Annual	PM2.5, PM10, O3, NO2, CO	Both	CVD (I00–I99)	All ages	Cfa	C-subtropical	H
35	Analitis et al.	2007	15 European cities	1990–2000	CC	Mean	NA (NA to NA)	NA	Annual	NA	Mortality	CVD (I00–I99)	All ages	Multi	Multi	H
36	Denpetkul and Phosri	2021	65 provinces, Thailand	2010–2017	TS	Mean	27.48 (6.95 to 36.6)	27.48	Annual	NA	Mortality	CVD (I00–I99)	All ages	Aw	A-tropical	UM
37	Guo et al.	2013	Five cities, China	2004–2008	TS	Mean	17.12 (−10.5 to 34.2)	17.12	Annual	PM10, NO2	Mortality	CVD (I00–I99)	All ages	NA	NA	UM
38	Chen et al.	2017	Texas, United States	1992–2011	CC	Cold spell	20.4 (−6.4 to 34.4)	20.4	Annual	NA	Mortality	CVD (I00–I99)	All ages	Dfa	D-continental	H
39	Zeka et al.	2014	Ireland	1984–2007	TS	Tapp	5.8 (3.1 to 8.6)	5.8	Cold (Dec–Feb)	NA	Mortality	CVD (I00–I99)	18+	Cfb	C-oceanic	H
40	Medina-Ramon and Schwartz	2007	50 cities, United States	1989–2000	CC	Min	19.8 (18.5 to 32.1)	19.8	Warm (May–Sep)	NA	Mortality	CHD (I20–I25)	All ages	NA	NA	H
41	Ha and Kim	2013	Seoul, South Korea	1993–2009	TS	Mean	24.4 (NA to NA)	24.4	Warm (Jun–Aug)	NA	Mortality	CVD (I00–I99)	All ages	Dwa	D-continental	H
42	Tian et al.	2012	Beijing, China	2000–2011	CC	Mean	13.3 (−7.6 to 30.5)	13.3	Annual	NA	Mortality	CHD (I20–I25)	All ages	Dwa	D-continental	UM
43	Sharafkhani et al.	2017	Urmia, Iran	2005–2010	TS	Mean	NA (NA to NA)	NA	Annual	PM10, NO2, SO2	Mortality	CVD (I00–I99)	All ages	Cfa	C-subtropical	UM
44	Pan et al.	1995	Taiwan, China	1981–1991	TS	Mean	NA (9 to 32)	NA	Annual	NA	Mortality	Stroke (I60–I69), CHD (I20–I25)	45+	Cfa	C-subtropical	H
45	Yang et al.	2015	Shanghai, China	1981–2012	TS	Mean	16.9 (−4.8 to 34.6)	16.9	Annual	NA	Mortality	CVD (I00–I99)	All ages	Cfa	C-subtropical	UM
46	Breitner et al.	2014	Bavaria, Germany	1990–2006	TS	Mean	9.5 (−14.2 to 29.2)	9.5	Annual	PM10, O3	Mortality	CVD (I00–I99)	All ages	Dfb	D-continental	H
47	Rodrigues et al.	2019	Lisbon, Portugal	2000–2013	TS	Mean	17.38 (4.1 to 33.3)	17.38	Annual	PM10	Mortality	Stroke (I60–I69)	All ages	Csa	C-mediterranean	H
48	Chen et al.	2014	Six cities, China	2009–2011	TS	Mean	4.9–23 (−24.2 to 33)	15	Annual	PM10, NO2, SO2	Mortality	CHD (I20–I25)	All ages	NA	NA	UM
49	Chan et al.	2012	Hong Kong, China	1998–2006	TS	Mean	27.6 (19.7 to 31.8)	27.6	Warm (May–Oct)	PM10, O3, NO2, SO2	Mortality	CVD (I00–I99)	All ages	Cfa	C-subtropical	UM
50	Rocklöv et al.	2011	Stockholm, Sweden	1990–2002	TS	Tappmin	21.6 (5.8 to 33.5)	21.6	Cold (Oct–Mar)	O3, NO2	Mortality	CVD (I00–I99)	All ages		E-subarctic	H
51	Fu et al.	2018	India	2001–2013	CC	Mean	NA (NA to NA)	NA	Annual	NA	Mortality	CVD (I00–I99)	All ages		C-subtropical	UM
52	Goodman et al.	2004	Dublin, Ireland	1980–1996	TS	Min	6.5 (−7.9 to 18.4)	6.5	Annual	PM10	Mortality	CVD (I00–I99)	All ages	Cfb	C-oceanic	H
53	Bai et al.	2014	Three cities, Tibet	2008–2012	TS	Mean	5.8–9.7 (−12.2 to 22.6)	7.5	Annual	NA	Mortality	CVD (I00–I99)	All ages	Dw(x)	D-continental	UM
54	Liu et al.	2011	Beijing, China	2003–2005	TS	Mean	22.6 (6.9 to 32.1)	22.6	Cold (Oct–Mar)	PM2.5	Mortality	CVD (I00–I99)	All ages	Dwa	D-continental	UM
55	Alahmad et al.	2020	Kuwait	2010–2016	TS	Mean	27.9 (6.86 to 44.65)	27.9	Annual	PM10, O3	Mortality	CVD (I00–I99)	All ages	BWh	B-dry	H
56	Iranpour et al.	2020	Ahvaz, Iran	2014–2018	TS	Mean	26.95 (5.8 to 42.4)	26.95	Annual	PM2.5, PM10, O3, NO2, CO, SO2	Mortality	CVD (I00–I99)	All ages	BSh	B-dry	UM
57	Yin et al.	2019	Beijing, China	2010–2016	TS	Mean	NA (−14 to 35)	NA	Annual	PM10	Mortality	CVD (I00–I99)	All ages	Dwa	D-continental	UM
58	Chen et al.	2018	272 cities, China	2013–2015	TS	Mean	15 (−0.5 to 25)	15	Annual	PM10, O3	Mortality	CVD (I00–I99)	All ages	NA	NA	UM
59	Hu et al.	2019	89 Zhejiang counties, China	2009–2015	TS	Mean	16.9 (−2 to 35.3)	16.9	Annual	PM10, O3	Mortality	CVD (I00–I99)	All ages	Multi	Multi	UM
60	Yang et al.	2015	15 cities, China	2007–2013	TS	Mean	5.3–21.6 (−28 to 36.7)	12	Annual	NA	Mortality	CVD (I00–I99)	All ages	Dwa	D-continental	UM
61	Chen et al.	2013	Eight cities, China	1996–2008	TS	Mean	16 (−22 to 34)	16	Annual	PM10, NO2, SO2	Mortality	Stroke (I60–I69)	All ages	Multi	Multi	UM
62	Zhang et al.	2021	Ganzhou, China	2015–2019	TS	Mean	20.4 (−3 to 39)	20.4	Annual	PM2.5, PM10, O3, NO2, CO, SO2	Mortality	CVD (I00–I99)	All ages	Cfa	C-subtropical	UM
63	Rocklov and Forsberg	2008	Stockholm, Sweden	1998–2003	TS	Mean	NA (NA to NA)	NA	Annual	NA	Mortality	CVD (I00–I99)	All ages	Cfb	E-subarctic	H
64	Yatim et al.	2021	Klang Valley, Malaysia	2006–2015	TS	Mean	27.7 (23.5 to 30.9)	27.7	Annual	PM10, O3	Mortality	CVD (I00–I99)	All ages	Af	A-tropical	UM
65	Xu et al.	2022	Jiangsu, China	2015–2019	CC	Mean	NA (3.2–27.8)	NA	Annual	PM2.5, PM10, O3, NO2, CO, SO2	Mortality	CVD (I00–I99)	All ages	Cfa	C-subtropical	UM
66	Schlte et al.	2021	seven geographic regions in Switzerland	1998–2016	TS	Mean	22 (3 to 40)	22	Annual	NA	Both	CVD (I00–I99)	All ages		D-continental	H
67	Lu et al.	2021	Queensland, Australia	1997–2013	CC	Mean	23.4 (−8.9 to 48.3)	23.4	Annual	NA	Mortality	CVD (I00–I99)	All ages	Multi	Multi	H
68	Wang et al.	2015	two cities, China	2007–2009	TS	Mean	15.65 (−9.4 to 34.6)	15.65	Annual	PM10, NO2, SO2	Mortality	CVD (I00–I99)	All ages	Dwa	D-continental	UM
69	Polcaro-Pichet	2019	Quebec, Canada	1981–2015	CC	Mean	NA (NA to NA)	NA	Cold (Nov–Apr)	NA	Mortality	Stroke (I60–I69)	All ages	Dfb	D-continental	H
70	Klot et al.	2012	48 cities in the United States	1992–2000	CC	Mean	NA (NA to NA)	NA	Cold (winter)	NA	Mortality	CVD (I00–I99)	All ages	Multi	Multi	H
71	Moghadamnia et al.	2018	Rasht, Iran	2005–2014	TS	Tapp	17.38 (−2.6 to 38.6)	17.38	Annual	NA	Mortality	CVD (I00–I99)	All ages	Cfa	C-subtropical	UM
72	Breitner et al.	2014	Three regions, Germany	1990–2006	TS	Mean	9.5 (−15.3 to 28.7)	9.5	Cold (Dec–Feb)	PM10, O3	Mortality	CVD (I00–I99)	All ages	Cfb	C-oceanic	H
73	Chen et al.	2019	Augsburg, Germany	1987–2014	CC	Mean	9.6 (−5.5 to 23.5)	9.6	Annual	PM10, O3, NO2	Mortality	CHD (I20–I25)	All ages	Cfb	C-oceanic	H
74	Nafstad et al.	2001	Oslo, Norway	1990–1995	TS	Mean	12.5 (NA to NA)	12.5	Warm (Apr–Sep)	NO2	Mortality	CVD (I00–I99)	All ages	Dfb	E-subarctic	H
75	Zhang et al.	2018	Yinchuan, China	2010–2015	TS	Mean	10.5 (−15 to 30.6)	10.5	Annual	NA	Mortality	CVD (I00–I99)	All ages	BSk	B-dry	UM
76	Gholampour et al.	2019	Isfahan, Iran	2008–2016	TS	Mean	17.52 (−7.4 to 35.4)	17.52	Annual	NA	Mortality	CVD (I00–I99)	All ages	BWk	B-dry	UM
77	Tsoutsoubi et al.	2021	Greece	1999–2012	TS	Mean	NA（−3 to 42）	NA	Annual	NA	Mortality	CVD (I00–I99)	70+	Multi	Multi	H
78	Kim et al.	2015	Seoul, South Korea	1995–2011	TS	Mean	12.8 (−15.7 to 30.4)	12.8	Annual	PM10	Mortality	CVD (I00–I99)	All ages	Dwa	D-continental	H
79	Anderson and Bell	2009	107 communities, United States	1987–2000	TS	Mean	NA (NA to NA)	NA	Annual	PM10, O3	Mortality	CVD (I00–I99)	All ages	Multi	Multi	H
80	Saucy et al.	2021	Zurich, Switzerland	2000–2015	TS	Mean	9 (−14 to 28)	9	Annual	PM2.5, NO2	Mortality	CVD (I00–I99)	All ages	Cfb	C-oceanic	H
81	Ma et al.	2021	47 prefectures, Japan	1972–2015	CC	Cold spell	NA (−0.5 to 18.6)	NA	Cold (Nov–Mar)	NA	Mortality	CVD (I00–I99)	All ages	Multi	Multi	UM
82	Ebi et al.	2004	Three regions, United States	1983–1998	TS	Min	NA (NA to NA)	NA	Annual	NA	Morbidity	CVD (I00–I99)	55+	Csa	C-mediterranean	H
83	Rocklov et al.	2014	Stockholm, Sweden	1990–2002	TS	Cold spell	NA (NA to NA)	NA	Annual	NO2	Morbidity	CVD (I00–I99)	All ages		E-subarctic	H
84	Wang et al.	2013	Jinan, China	1990–2009	TS	Mean	15 (−10.5 to 35.8)	15	Annual	NA	Morbidity	Stroke (I60–I69)	All ages	Cwa	C-subtropical	UM
85	Hajat et al.	2002	London, United Kingdom	1992–1995	CC	Mean	8.1 (NA to NA)	8.1	Cold (Oct–Mar)	SO2, O3, PM10	Morbidity	CVD (I00–I99)	65+	Cfb	C-oceanic	H
86	Shaposhnikov et al.	2014	Moscow, Russia	1992–2005	TS	Mean	5.5 (−17 to 25)	5.5	Annual	NA	Morbidity	Stroke (I60–I69)	All ages	Dfb	D-continental	UM
87	Kovats et al.	2004	London, United Kingdom	1994–2000	TS	Mean	11.6 (3.1 to 26.7)	11.6	Annual	PM10, O3	Morbidity	CVD (I00–I99)	All ages	Cfb	C-oceanic	H
88	Wu et al.	2011	Taiwan, China	1994–2003	CC	Cold spell	NA (NA to NA)	NA	Cold (Nov–Jan)	NA	Mortality	CVD (I00–I99)	All ages	Cfa	C-subtropical	UM
89	Kysely et al.	2009	Czech Republic	1994–2006	CC	Cold spell	NA (−1.9 to 21.9)	NA	Cold (Dec–Feb)	NA	Mortality	CVD (I00–I99)	25+		C-oceanic	H
90	Madrigano et al.	2013	Worcester, United States	1995–2003	CC	Cold spell	7.9 (−6.9 to 25.5)	7.9	Annual	PM2.5, O3	Mortality	ACS (I21–I22)	All ages	Dfa	D-continental	H
91	Lu et al.	2020	Queensland, Australia	1995–2016	CC	Mean	25.9 (−8.9 to 48.8)	25.9	Annual	NA	Morbidity	CVD (I00–I99)	All ages	Multi	Multi	H
92	Bai et al.	2018	Ontario, Canada	1996–2013	TS	Mean	NA (−33.1 to 32.2)	NA	Annual	PM10, NO2, PM2.5	Morbidity	CVD (I00–I99)	All ages	Dfb	D-continental	H
93	Chen et al.	2010	Taiwan, China	1997–2003	TS	Cold spell	NA (NA to NA)	NA	Annual	NA	Mortality	CVD (I00–I99)	All ages	Cfa	C-subtropical	UM
94	Martinez-Solanas and Basagana	2017	Spain	1997–2013	TS	Max	20.9 (6.88 to 34.69)	20.9	Annual	NA	Morbidity	CVD (I00–I99)	All ages	Multi	Multi	H
95	Mohammad et al.	2020	Hong Kong, China	1998–2011	TS	Mean	23.52 (8.2 to 31.8)	23.52	Annual	PM10, NO2, O3	Morbidity	CVD (I00–I99)	All ages	Cfa	C-subtropical	H
96	Ryti et al.	2017	Oulu, Finland	1998–2011	CC	Cold spell	1.4 (−41.3 to 33)	1.4	Annual	NA	Mortality	CVD (I00–I99)	All ages		E-subarctic	H
97	Ryti et al.	2018	Oulu, Finland	1998–2011	CC	Cold spell	1.4 (−41.3 to 33)	1.4	Annual	NA	Mortality	Atherosclerotic heart disease (I25.1)	35+		E-subarctic	H
98	Sartini et al.	2016	London, United Kingdom	1998–2012	TS	Cold spell	NA (NA to NA)	NA	Annual	NA	Mortality	CVD (I00–I99)	60+	Cfb	C-oceanic	H
99	Wichmann et al.	2012	Copenhagen, Denmark	1999–2006	CC	TappMax	16 (0 to 30)	16	Annual	PM10, NO2, CO	Morbidity	ACS (I21–I22)	18+	Cfb	C-oceanic	H
100	Wang and Lin	2014	Taipei, China	2000 –2009	TS	Mean	23.4 (8.3 to 33)	23.4	Annual	PM10, NO2, O3	Morbidity	CVD (I00–I99)	All ages	Cfa	C-subtropical	UM
101	Liang et al.	2008	Taichung, China	2000–2003	TS	Mean	27–29 (NA to NA)	28	Annual	PM10, NO2, CO, SO2, O3	Morbidity	ACS (I21–I22)	All ages	Cfa	C-subtropical	H
102	Revich and Shaposhnikov	2008	Moscow, Russia	2000–2006	CC	Cold spell	NA (NA to NA)	NA	Annual	NA	Mortality	CHD (I20–I25), stroke (I60–I69)	All ages	Dfb	D-continental	H
103	Dahlquist et al.	2016	Stockholm, Sweden	2000–2010	CC	Mean	7.1 (−18.2 to 25.2)	7.1	Annual	PM10, O3	Morbidity	OHCA (I46)	All ages	Cfb	C-oceanic	H
104	Lin et al.	2021	Five cities, Taiwan, China	2000–2014	TS	Mean	23.1–25.4 (NA to NA)	14	Annual	PM10, O3, NO2, SO2	Morbidity	CVD (I00–I99)	40+	Cfa	C-subtropical	H
105	Vaičiulis et al.	2021	Kaunas, Lithuanian	2000–2015	TS	Cold spell	NA (NA to NA)	NA	Cold (Nov–Jan)	NA	Mortality	MI (I21–I23)	25+		D-continental	H
106	Ma et al.	2013	Shanghai, China	2001–2009	TS	Cold spell	17.5 (−3.4 to 39)	17.5	Cold (Jan–Mar)	NA	Mortality	CVD (I00–I99)	All ages	Dwa	D-continental	UM
107	Wichmann et al.	2011	Greater Copenhagen, Denmark	2002–2006	CC	Tapp	10 (−8 to 30)	10	Cold (Oct–Mar)	PM10, NO2, CO	Morbidity	CVD (I00–I99)	All ages	Cfb	C-oceanic	H
108	Goggins et al.	2017	Hong Kong, China	2002–2011	TS	Mean	23.4 (8.2 to 31.8)	23.4	Annual	PM10, O3	Morbidity	CVD (I00–I99)	0–59 ages	Cfa	C-subtropical	H
109	Kim et al.	2021	Seven metropolitan provinces, South Korea	2002–2017	TS	Mean	NA (NA to NA)	NA	Annual	PM2.5, PM10, O3, NO2, CO, SO2	Morbidity	ACS (I21–I22)	All ages	Multi	Multi	H
110	Misailidou et al.	2006	Five rural regions, Greece	2003–2004	TS	Mean	NA (NA to NA)	NA	Annual	NA	Morbidity	ACS (I21–I22)	All ages	Multi	Multi	H
111	Vasconcelos et al.	2013	Lisbon and Oporto, Portugal	2003–2007	TS	Mean	NA (NA to NA)	NA	Cold (winter)	PM10	Morbidity	MI (I21–I23)	All ages	Csa	C-mediterranean	H
112	Son et al.	2014	Eight cities, South Korea	2003–2008	TS	Mean	14.1 (12.6 to 16.2)	14.1	Annual	NA	Morbidity	CVD (I00–I99)	All ages	Multi	Multi	H
113	Ma et al.	2011	Shanghai, China	2005–2008	TS	Cold spell	17.7 (−3.1 to 34.1)	17.7	Cold (Jan–Feb)	NA	Morbidity	CVD (I00–I99)	All ages	Cfa	C-subtropical	UM
114	Cho et al.	2018	Seoul, South Korea	2005–2009	TS	Mean	12.9 (−11.5 to 30.1)	12.9	Annual	O3, PM2.5	Morbidity	Stroke (I60–I69)	All ages	Dwa	D-continental	H
115	Yamazaki and Michikawa	2017	Three prefectures, Japan	2005–2012	CC	Mean	16.83 (NA to NA)	16.83	Annual	NA	Morbidity	OHCA (I46)	All ages	Cfa	C-subtropical	H
116	Bai et al.	2014	Lhasa, Tibet, China	2005–2012	TS	Max	9.6 (−16.1 to 30.4)	9.6	Annual	NA	Morbidity	CVD (I00–I99)	All ages	Dwb	D-continental	UM
117	Tian et al.	2016	Hong Kong, China	2005–2012	CC	Mean	23.4 (8.7 to 31.8)	23.4	Annual	NO2, PM10, O3	Morbidity	CVD (I00–I99)	All ages	Cfa	C-subtropical	UM
118	Xu et al.	2021	Brisbane, Australia	2005–2013	CC	Mean	NA (NA to NA)	NA	Annual	PM10, NO2	Morbidity	Stroke (I60–I69)	All ages	Cfa	C-subtropical	H
119	Moghadamnia et al.	2018	Rasht, Iran	2005–2014	TS	Tapp	17.4 (−2.6 to 38.6)	17.4	Annual	NA	Morbidity	ACS (I21–I22)	All ages	Cfa	C-subtropical	UM
120	Onozuka et al.	2017	Japan	2005–2014	TS	Mean	9.4–23.2 (−10.7 to 33.7)	16	Annual	NA	Morbidity	OHCA (I46)	All ages	Multi	Multi	H
121	Shin et al.	2021	Seoul, South Korea	2005–2014	CC	Min	12.7 (NA to NA)	12.7	Annual	NO2, CO, SO2	Morbidity	MI (I21–I23)	All ages	Dwa	D-continental	H
122	Cheng et al.	2020	Brisbane, Australia	2005–2015	TS	Mean	16 (10 to 25)	16	Annual	PM10, NO2,	Morbidity	MI (I21–I23)	All ages	Cfa	C-subtropical	H
123	Zhou et al.	2014	15 provinces in China	2006–2010	CC	Cold spell	NA (−1.9 to 21.9)	NA	Cold (Dec–Feb)	NA	Mortality	CVD (I00–I99)	All ages	Cfa	C-subtropical	UM
124	Ponjoan et al.	2017	Two regions, Spain	2006–2013	CC	Cold spell	28.1 (27.3 to 29)	28.1	Cold (Nov–Jan)	PM10, O3, NO2, SO2, CO	Morbidity	CVD (I00–I99)	All ages	Multi	Multi	H
125	Lee et al.	2014	16 cities, South Korea	2006–2014	TS	Mean	13.3 (−19.5 to 37.7)	13.3	Annual	PM10, NO2, CO, SO2, O3，PM2.5	Morbidity	ACS (I21–I22)	All ages	Multi	Multi	H
126	Wang et al.	2020	Taiwan, China	2006–2014	TS	Mean	23.4 (10.6 to 31)	23.4	Annual	PM10,NO2, PM2.5	Morbidity	OHCA (I46)	All ages	Cfa	C-subtropical	H
127	Moraes et al.	2022	São Paulo, Brazil	2006–2015	CC	Cold spell	NA (NA to NA)	NA	Annual	PM10	Mortality	CVD (I00–I99)	All ages	Cfa	C-subtropical	UM
128	Chen et al.	2019	31 cities, China	2007–2013	CC	Cold spell	NA (NA to NA)	NA	Annual	NA	Mortality	CVD (I00–I99)	All ages	Cfa	C-subtropical	UM
129	Doan et al.	2021	Brisbane, Australia	2007–2019	TS	Mean	20.9 (10.4 to 30.1)	20.9	Annual	NA	Morbidity	OHCA (I46)	All ages	Cfa	C-subtropical	H
130	Giang et al.	2014	Thai Nguyen, Vietnam	2008–2012	TS	Mean	23.6 (21 to 27.5)	23.6	Annual	NA	Morbidity	CVD (I00–I99)	60+	Cfa	C-subtropical	LM
131	Niu et al.	2016	Guangzhou, China	2008–2013	TS	Mean	22.3 (5.1 to 33.5)	22.3	Annual	PM10, NO2, SO2	Morbidity	OHCA (I46)	All ages	Cfa	C-subtropical	UM
132	Thu Dang et al.	2019	Two Central Coast regions, Vietnam	2008–2015	TS	Mean	26.1 (15.0 to 36.9)	26.1	Annual	NA	Morbidity	ACS (I21–I22)	All ages	NA	NA	LM
133	Bijelović et al.	2017	Novi Sad, Serbia	2010–2011	TS	Mean	NA (NA to NA)	NA	Annual	NA	Morbidity	ACS (I21–I22)	19+	Cfa	C-subtropical	UM
134	Sangkharat et al.	2020	London, United Kingdom	2010–2014	TS	Mean	11.8 (−2.2 to 25.4)	11.8	Annual	PM10, NO2, CO, SO2, O3, PM2.5	Morbidity	CVD (I00–I99)	All ages	Cfb	C-oceanic	H
135	Hensel et al.	2017	Hamburg, Germany	2010–2014	TS	Mean	10 (NA to NA)	10	Annual	NA	Morbidity	CVD (I00–I99)	All ages	Cfb	C-oceanic	H
136	Pourshaikhian et al.	2019	Rasht, Iran	2010–2015	TS	Tapp	30.1 (NA to NA)	30.1	Warm (May–Sep)	NA	Morbidity	CVD (I00–I99)	All ages	Cfa	C-subtropical	UM
137	Zhan et al.	2022	Fujian province, China	2010–2016	TS	TappMean	20 (−2 to 33.8)	20	Annual	PM10, NO2, CO, SO2	Morbidity	CVD (I00–I99)	All ages	Cfa	C-subtropical	UM
138	Han et al.	2017	Jinan, China	2011–2014	TS	Cold spell	14.7 (−9.4 to 34)	14.7	Annual	NA	Mortality	CVD (I00–I99)	All ages	Cwa	C-subtropical	UM
139	Mohammadi et al.	2021	Sabzevar, Iran	2011–2017	TS	Tapp	12.9 (−11.2 to 45.4)	12.9	Annual	NA	Morbidity	CVD (I00–I99)	All ages	DSk	D-continental	UM
140	Zhao et al.	2018	Ningxia Hui Autonomous Region, China	2012–2015	TS	Mean	8.5 (−18.6 to 29.7)	8.5	Annual	NO2, CO, SO2, PM2.5	Morbidity	CVD (I00–I99)	All ages	BWk	B-dry	UM
141	Luo et al.	2017	Beijing, China	2013–2014	TS	Mean	11.6 (−12.9 to 30.1)	11.6	Annual	PM2.5	Morbidity	Stroke (I60–I69)	All ages	Cfa	C-subtropical	UM
142	Guo et al.	2017	Guangzhou, China	2013–2015	TS	Mean	NA (NA to NA)	NA	Annual	NO2, SO2, O3, PM2.5	Morbidity	Stroke (I60–I69)	All ages	Cfa	C-subtropical	UM
143	Gao et al.	2019	Hefei, China	2013–2015	TS	Cold spell	NA (NA to NA)	NA	Annual	NO2, PM10, O3	Morbidity	CVD (I00–I99)	All ages	Cfa	C-subtropical	UM
144	Lei et al.	2022	272 cities, China	2013–2015	CC	Mean		NA	Annual	PM2.5, O3	Mortality	CVD (I00–I99)	All ages	Cfa	C-subtropical	UM
145	Liu et al.	2018	Beijing, China	2013–2016	TS	Mean	12.8 (−16 to 32)	12.8	Annual	NA	Morbidity	ACS (I21–I22)	All ages	Dwa	D-continental	UM
146	Aklilu et al.	2020	Beijing, China	2013–2017	CC	Mean	13.9 (−14.1 to 32.6)	13.9	Annual	PM10, NO2, CO, SO2, O3, PM2.5	Morbidity	CVD (I00–I99)	All ages	Dwa	D-continental	UM
147	Garcı´a-Lledó et al.	2020	Madrid, Spain	2013–2017	TS	Max	NA (NA to NA)	NA	Annual	NA	Morbidity	ACS (I21–I22)	All ages	BSk	B-dry	H
148	Guo et al.	2020	Yancheng, China	2013–2018	TS	Mean	15.2 (−4.7 to 32.9)	15.2	Annual	PM2.5, O3, NO2, CO, SO2	Morbidity	ACS (I21–I22)	All ages	Cfa	C-subtropical	UM
149	Wang et al.	2021	Qingdao, China	2014–2017	TS	Mean	14.9 (NA to NA)	14.9	Annual	PM10, PM2.5, SO2, NO2, CO, O3	Morbidity	CVD (I00–I99)	All ages	Cfa	C-subtropical	UM
150	Wang et al.	2021	Shenzhen, China	2015–2016	TS	Mean	23.5 (NA to NA)	23.5	Warm (May–Oct)	SO2, O3, PM2.5	Morbidity	CVD (I00–I99)	All ages	Cfa	C-subtropical	UM
151	Cui et al.	2019	Hefei, China	2015–2017	TS	Mean	18.1 (−5.9 to 35.6)	18.1	Annual	PM10, NO2, SO2	Morbidity	CVD (I00–I99)	All ages	Cfa	C-subtropical	UM
152	Mohammad et al.	2018	Sweden	2017–2018	TS	Min	NA (−11.1 to 11.5)	NA	Annual	NO2, CO, O3, PM2.5	Morbidity	CHD (I20–I25)	All ages	Dfb	E-subarctic	H
153	Li et al.	2021	Beijing, China	2017–2019	TS	Tapp	10 to 12 (−6 to 33)	11	Annual	PM10, NO2, CO, SO2, O3, PM2.5	Morbidity	ACS (I21–I22)	All ages	Dwa	D-continental	UM
154	Borghei et al.	2020	Rasht, Iran	3-year period	TS	TappMean	17.2 (NA to NA)	17.2	Annual	NA	Morbidity	OHCA (I46)	All ages	Cfa	C-subtropical	UM
155	Li et al.	2017	Shenyang, China	2006–2015	TS	Mean	8.2 (−24.0 to 29)	8.2	Annual	PM10, NO2, SO2	Morbidity	DVT (I82)	All ages	Dwa	D-continental	UM
156	Chiara et al.	2021	8,084 municipalities of Italy	2006–2015	CC	Mean	13.7 (−25.8 to 38.5)	13.7	Annual	PM2.5, PM10	Morbidity	DVT (I82)	All ages	Cfb	C-oceanic	H
157	Chen et al.	2022	11 cities, China	2009–2019	CC	Mean	NA (NA to NA)	NA	Annual	PM2.5, O3	Morbidity	AAD (I71)	All ages	Cfa	C-subtropical	UM
158	Yu et al.	2021	Wuhan, China	2011–2018	TS	Mean	NA (NA to NA)	NA	Annual	SO2, NO2	Morbidity	AAD (I71)	All ages	Cfa	C-subtropical	UM
159	Zhang et al.	2022	131 cities, China	2015–2020	CC	Mean	NA (NA to NA)	NA	Annual	PM2.5, O3, NO2, CO, SO2	Morbidity	AAD (I71)	All ages	Cfa	C-subtropical	UM

T-S, time series; C-C, case-crossover; Min, minimum temperature; Max, maximum temperature; Mean, mean temperature; Tapp, apparent temperature; D, daily resolution; W, weekly resolution; M, monthly resolution; ACS, acute coronary syndrome; OHCA, out-of-hospital cardiac arrest; H, high-income; UM, upper-middle income; LM, lower-middle income; NA, not available.

### 2.4. Study quality assessment

We further appraised the evidence included in the meta analysis by applying the risk of bias (RoB) assessment in each study and assessment of quality and strength of the body of included studies. A detailed description of the criteria for the assessments is provided in Supplementary Tables S3–S5.

### 2.5. Statistical analysis

A random-effects meta-analysis was used to compute the relative risk (RR) estimates associated with cold exposure. We converted all RRs to RRs associated with a 1°C decrease below the reference temperature points, assuming a log-linear relationship between mortality/morbidity and temperature below the reference temperature points (6, 19). If studies reported multiple lag RRs, we selected the lag associated with the maximum risk to conduct the meta-analysis. Subgroup analysis was carried out to analyze the vulnerabilities stratified by age, sex, national income level, and climate zones (classified by the Köppen–Geiger climate zones) (20). Subgroup interaction was employed to detect the significance of differences among subgroups.

*I*^2^ statistics and Cochrane Q were used to examine heterogeneity among effect estimates. The heterogeneity of pooled estimates with *p* < 0.10 (Cochrane Q test) was deemed significant (21). *I*^2^ statistics of 0%–25%, 25%–50%, and >50% indicated low, moderate, and high heterogeneity, respectively (21). Funnel plots and Egger's test were used to evaluate potential publication bias, and the Trim and Fill method was used to examine the sensitivity of the results to publication bias. Sensitivity analyses were carried out, separating studies by temperature metrics, study design (time-series or case-crossover), seasonality, lag effects, and air pollution adjustment for low-temperature exposure, and intensity (classified by cold spell duration) for cold spells. We further examined the influence of individual estimates on the pooled RRs using a leave-one-out analysis. Meta-regressions were used further to explore the heterogeneity of effects and determinants of heterogeneity. Statistical analyses were done using Stata (version 16.0).

## 3. Results

### 3.1. Search and study selection results

The initial database searches produced a total of 21,724 articles, of which 1,672 duplicate records were excluded. After screening titles and abstracts, 19,773 articles were eliminated for being irrelevant. We included 166 articles for full-text review and excluded 7: 1 because of an overlapping database (22) and 6 for providing non-standardizable estimates (23–28). Ultimately, we identified 159 studies on the basis of the inclusion criteria for the final review (Figure 1).

### 3.2. Study characteristic

**Figure 2:**
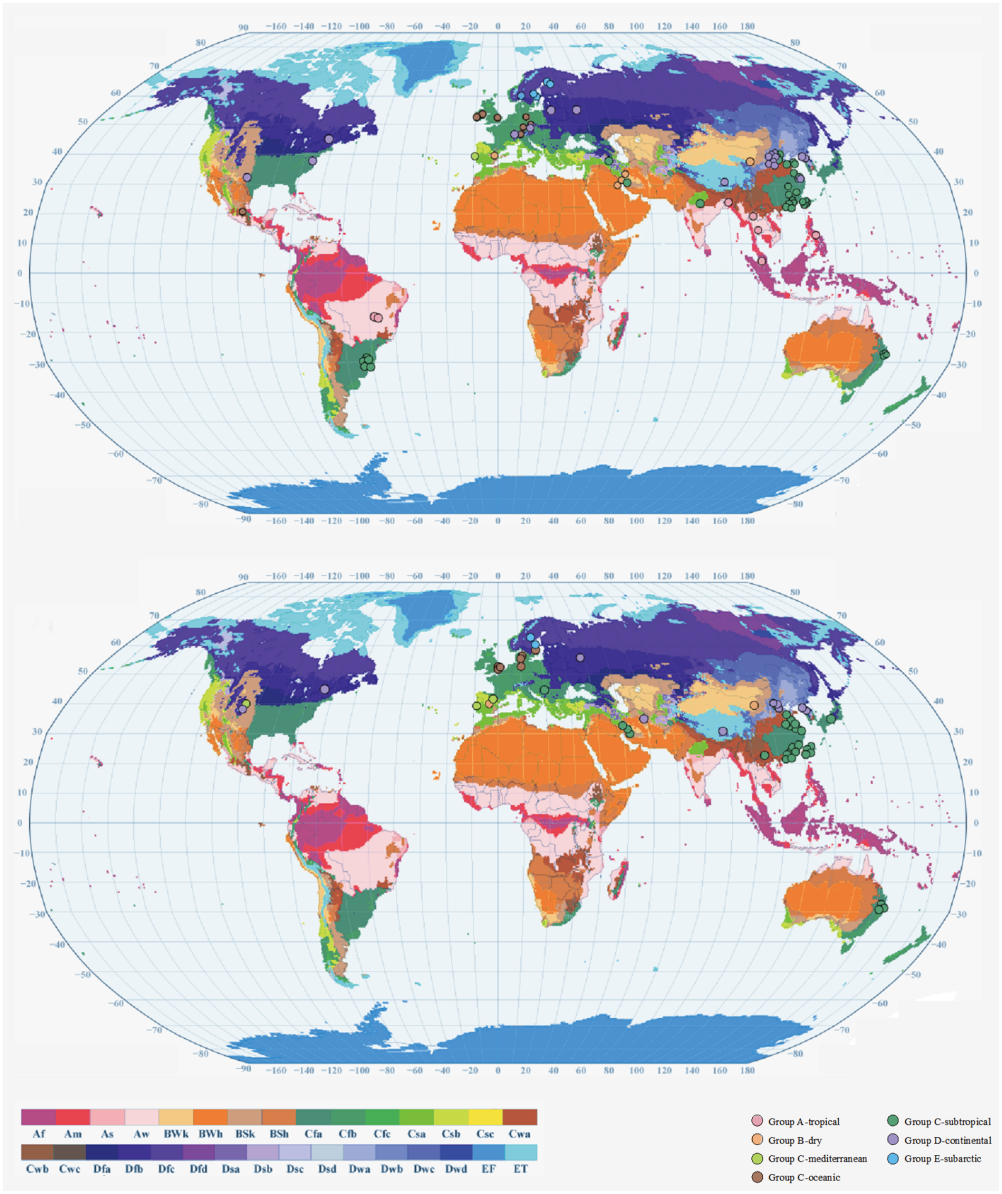
Geographical distribution of city-specific or region-specific cardiovascular disease mortality (**A**) and cardiovascular disease morbidity (**B**) estimates included in the meta-analysis by considering the Köppen–Geiger climate zone. Af, tropical rainforest climate; Am, tropical monsoon climate. Aw, tropical savanna climate. BWh, hot desert climate; BWk, cold desert climate; BSh, hot semiarid climate; BSk, cold semiarid climate; Csa, hot-summer Mediterranean climate; Csb, warm-summer Mediterranean climate; Csc, cold-summer Mediterranean climate; Cwa, monsoon-influenced humid subtropical climate; Cwb, subtropical highland climate or monsoon-influenced temperate oceanic climate; Cwc, cold subtropical highland climate or monsoon-influenced subpolar oceanic climate; Cfa, humid subtropical climate; Cfb, temperate oceanic climate; Cfc, subpolar oceanic climate; Dsa, Mediterranean-influenced hot-summer humid continental climate; Dsb, Mediterranean-influenced warm-summer humid continental climate; Dsc, Mediterranean-influenced subarctic climate; Dsd, Mediterranean-influenced extremely cold subarctic climate; Dwa, monsoon-influenced hot-summer humid continental climate; Dwb, monsoon-influenced warm-summer humid continental climate; Dwc, monsoon-influenced subarctic climate; Dwd, monsoon-influenced extremely cold subarctic climate; Dfa, hot-summer humid continental climate; Dfb, warm-summer humid continental climate; Dfc, subarctic climate; Dfd, extremely cold subarctic climate; ET, tundra climate; EF, ice cap climate.

The characteristics of the included studies presented in Table 1. Of these, 135 studies reported low-temperature effects, 21 assessed cold spell effects, and 2 examined the effects of both low temperature and cold spells. A total of 80 studies reported cardiovascular mortality, 64 studies assessed morbidity, and 4 reported both health outcomes. According to the Köppen–Geiger climate zones classification (20), 7 studies were carried out in the tropical zone, 7 in the dry zone, 5 in the Mediterranean zone, 16 in the oceanic climate zone, 63 in the subtropical zone, 29 in the continental area, and 7 in the subarctic zone (Figure 2).

### 3.3. Meta-analysis of low-temperature effects

**Figure 3:**
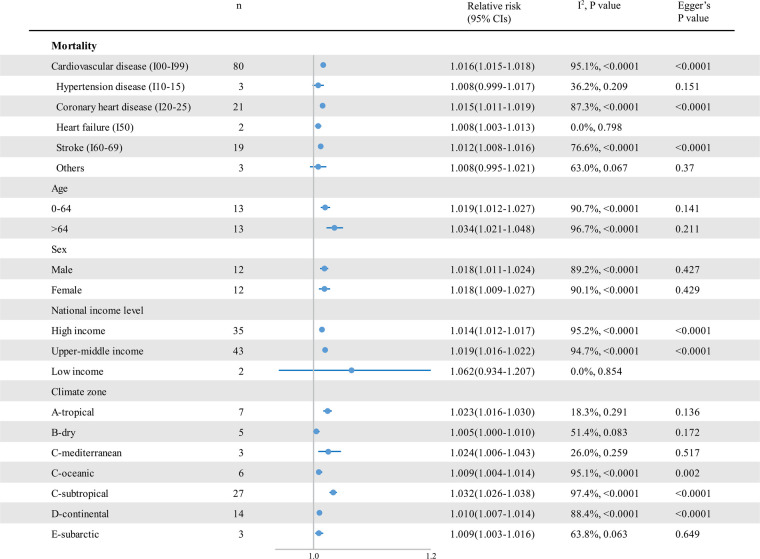
The impact of low temperatures on RR and 95% CIs for cardiovascular disease mortality in different groups. RR, relative risk; *n*, the number of effect estimates; CI, confidence interval.

**Figure 4:**
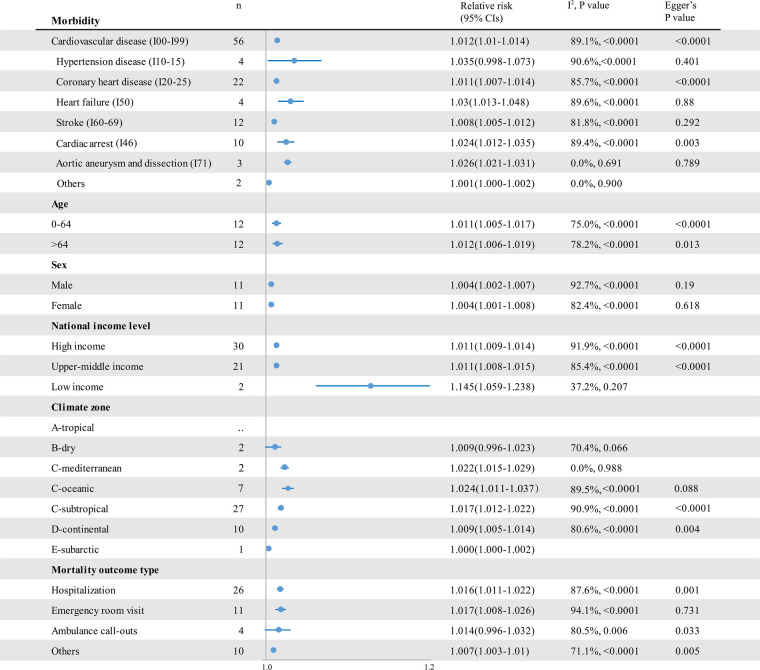
The impact of low temperatures on RR and 95% CIs for cardiovascular disease morbidity in different groups. RR, relative risk; *n*, the number of effect estimates; CI, confidence interval.

An analysis of pooled estimates showed that for every 1°C decrease in temperature, cardiovascular disease–related mortality increased by 1.6% [RR 1.016; 95% confidence interval (CI) 1.015–1.018] (Figure 3 and Supplementary Figure S1), and cardiovascular morbidity increased by 1.2% (RR 1.012; 95% CI 1.010–1.014) (Figure 4 and Supplementary Figure S2). Cause-specific analyses showed positive associations between low temperatures and the mortality of coronary heart disease (CHD) (RR 1.015; 95% CI 1.011–1.019), heart failure (HF) (RR, 1.008; 95% CI 1.003–1.013), and stroke (RR 1.012; 95% CI 1.008–1.016), while cold temperatures showed no significant association with hypertensive diseases and cardiac arrest mortality. For morbidity, low temperatures increased the morbidity of all kinds of cardiovascular diseases, apart from hypertensive diseases. Moreover, higher morbidity risks were attributable to HF (RR 1.030; [95% CI 1.013–1.048]), aortic aneurysm and dissection (AAD) (RR 1.026; 95% CI 1.021–1.031), and out-of-hospital cardiac arrest (RR 1.024; 95% CI 1.012–1.035). We further analyzed the cold effects in a population with different characteristics to explore the population's vulnerability. We found that people aged 65 or older were more vulnerable to cardiovascular disease–related mortality (*p* = 0.056). Considering the climate zones, a significant greater risk of cardiovascular disease–related mortality was observed in those who lived in tropical (*p* = 0.004) and subtropical (*p* < 0.001) climate zones than those in the subarctic climate zone. In addition, cardiovascular morbidity was significantly higher in people living in lower-middle-income countries than in those living in high-income and upper-middle-income countries (*p* = 0.002).

### 3.4. Meta-analysis of cold spell effects

**Figure 5:**
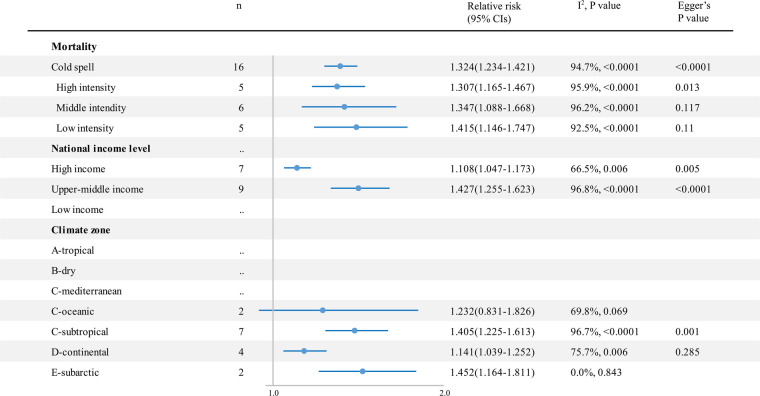
The impact of cold spells on RR and 95% CIs for cardiovascular disease mortality in different groups. RR, relative risk; *n*, the number of effect estimates; CI, confidence interval.

**Figure 6:**
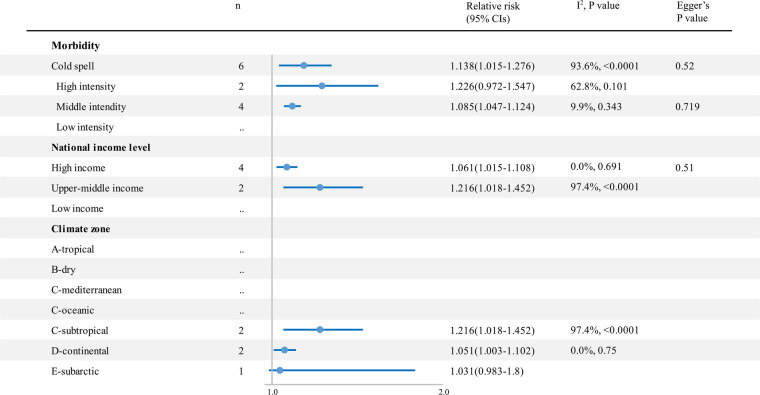
The impact of cold spells on RR and 95% CIs for cardiovascular disease morbidity in different groups. RR, relative risk; *n*, the number of effect estimates; CI, confidence interval.

Cold spells had a significant impact on cardiovascular outcomes, which increased cardiovascular disease–related mortality by 32.4% (RR 1.324; 95% CI 1.234–1.421) (Figure 5 and Supplementary Figure S3) and morbidity by 13.8% (RR 1.138; 95% CI 1.015–1.276) (Figure 6 and Supplementary Figure S4). There was no significant difference among cold spells of different intensities. Moreover, in the subarctic climate zone (RR 1.452; 95% CI 1.164–1.811), the effect of the cold spell on cardiovascular mortality was significantly higher than that in the continental area (*p* = 0.049).

### 3.5. Heterogeneity analysis

We found high heterogeneity in the summary effect estimates of low temperature (heterogeneity *p*-values < 0.0001, and all *I*^2^ > 50%). The stratification by sex and age did not help reduce heterogeneity, while it decreased in hypertensive disease and HF mortality stratum (Figure 3). We further conducted sensitivity analyses, finding no significant differences in the pooled RRs for the associations between cold exposure and cardiovascular disease–associated health outcomes in the leave-one-out analysis (low-temperature mortality RR 1.015–1.018; low-temperature morbidity RR 1.011–1.013). Moreover, for cardiovascular disease–related mortality and morbidity, a series of sensitivity analyses done by separating studies by temperature metrics, study design, seasonality, lag effects, and air pollution adjustment showed consistency in the direction and magnitude of the associations in the reviewed studies (Table 2). We further explored the source of heterogeneity using meta-regression (Supplementary Table S6), which showed that the lower-middle-income level was positively correlated with a 1% decrease in RRs for cold effects on cardiovascular morbidity (RR 1.124; 95% CI 1.035–1.221; *p* = 0.007; ref = high income level). The heterogeneity in the summary effect estimates of cold spells was large (heterogeneity *p*-values < 0.0001, and all *I*^2^ > 90%). The stratification of cold spell intensity only reduced the heterogeneity of the estimated morbidity RRs. No significant difference in the pooled RRs for the associations between cold spells and cardiovascular mortality was found in the leave-one-out analysis (cold spell morbidity RR 1.279–1.372). However, two (33%) studies could render the pooled effects of cold spells on cardiovascular disease morbidity insignificant when left out from the analysis.

**Table 2:** Sensitivity analysis of random-effects meta-analysis showing relative risk (RR) and 95% confidence intervals (CIs), for the association between low temperatures and cardiovascular disease morbidity, with every 1°C decrease in temperature.

	k	RR	lci	uci	*I* ^2^	*p*	Eagger's *p*-value
Adjusted for air pollution							
PM_2.5_ mortality	10	1.021	1.012	1.031	95.90%	<0.0001	0.013
PM_2.5_ morbidity	16	1.010	1.007	1.014	91.80%	<0.0001	0.005
PM_10_ mortality	40	1.024	1.019	1.028	96.40%	<0.0001	<0.0001
PM_10_ morbidity	21	1.020	1.013	1.028	91.40%	<0.0001	0.001
O_3_ mortality	28	1.028	1.021	1.035	96.90%	<0.0001	0.003
O_3_ morbidity	19	1.028	1.012	1.027	92.10%	<0.0001	0.002
NO_2_ mortality	31	1.026	1.019	1.032	95.80%	<0.0001	0.001
NO_2_ morbidity	22	1.011	1.008	1.015	90.70%	<0.0001	0.001
CO mortality	8	1.025	1.010	1.040	96.60%	<0.0001	0.658
CO morbidity	5	1.019	1.008	1.030	87.20%	<0.0001	0.865
SO_2_ mortality	20	1.026	1.018	1.035	95.60%	<0.0001	0.068
SO_2_ morbidity	18	1.016	1.009	1.023	79.30%	<0.0001	0.001
Null mortality	28	1.011	1.009	1.014	91.10%	<0.0001	0.001
Null morbidity	21	1.007	1.005	1.009	89.10%	<0.0001	<0.0001
Exposure							
Tmean mortality	70	1.015	1.013	1.016	95.30%	<0.0001	<0.0001
Tmean morbidity	48	1.014	1.011	1.017	90.00%	<0.0001	<0.0001
Tmax mortality	—						
Tmax morbidity	3	1.022	0.998	1.046	83.00%	0.003	0.169
Tmin mortality	4	1.019	1.009	1.029	65.30%	0.034	0.001
Tmin morbidity	3	1.012	0.996	1.028	93.70%	<0.0001	0.306
Tapp mortality	5	1.015	1.005	1.025	93.40%	<0.0001	0.168
Tapp morbidity	9	1.003	1.000	1.006	49.80%	0.063	0.028
Season							
Annual mortality	66	1.017	1.015	1.019	95.70%	<0.0001	<0.0001
Annual morbidity	56	1.011	1.009	1.013	88.70%	<0.0001	<0.0001
Cold mortality	9	1.010	1.006	1.015	84.20%	<0.0001	0.025
Cold morbidity	5	1.041	1.011	1.071	92.50%	<0.0001	0.235
Warm mortality	6	1.019	1.015	1.024	0.00%	0.757	0.95
Warm morbidity	2	1.010	0.989	1.032	79.40%	0.028	—
Study design							
TS mortality	68	1.019	1.017	1.022	95.50%	<0.0001	<0.0001
TS morbidity	48	1.011	1.090	1.013	89.80%	<0.0001	<0.0001
CC mortality	12	1.007	1.005	1.008	83.20%	<0.0001	0.007
CC morbidity	14	1.016	1.009	1.022	91.50%	<0.0001	0.007
Lag days mortality (day)							
Cumulative 0–9	17	1.017	1.010	1.023	96.80%	<0.0001	0.142
Cumulative 10–19	15	1.017	1.012	1.022	88.70%	<0.0001	0.0002
Cumulative >20	10	1.025	1.017	1.034	96.00%	<0.0001	0.002
Single 0–9	13	1.022	1.015	1.028	96.90%	<0.0001	0.02
Single >10	8	1.022	1.013	1.032	92.00%	<0.0001	0.083
Lag days morbidity (day)							
Cumulative 0–9	7	1.005	1.001	1.009	74.50%	<0.0001	0.054
Cumulative 10–19	4	1.012	1.003	1.022	91.80%	<0.0001	0.628
Cumulative >20	12	1.043	1.029	1.058	81.30%	<0.0001	0.008
Single 0–9	11	1.015	1.009	1.02	66.70%	0.001	0.032
Single >10	4	1.003	1	1.005	72.20%	0.013	0.359
Risk of bias (mortality)							
Low	10	1.014	1.009	1.019	83.20%	<0.0001	0.007
Probably low	67	1.016	1.015	1.018	95.50%	<0.0001	<0.0001
Probably high	3	1.021	1.012	1.027	83.00%	0.003	0.169
Risk of bias (morbidity)							
Low	6	1.017	1.009	1.026	96.60%	<0.0001	0.658
Probably low	51	1.012	1.01	1.014	91.50%	<0.0001	<0.0001
Probably high	4	1.023	1.014	1.027	93.40%	<0.0001	0.216

RR, relative risk, CI, confidence interval; T-S, time series; C-C, case-crossover; Tmin, minimum temperature; Tmax, maximum temperature; Tmean, mean temperature; Tapp, apparent temperature.

### 3.6. Rob and study quality assessment

We assessed the RoB of the included studies and rated the overall RoB according to the key components such as exposure, outcome, and confounding bias. The details of the RoB assessment criteria and individual studies’ assessment are given in Supplementary Table S2 and Supplementary Figure S5. In summary, of the 154 (96%) studies that were rated with low risk or probably with a low risk of bias, 8 (4%) were rated with probably high risk, and no study was rated with a high risk of overall bias (Supplementary Figure S6). The initial quality rating was moderate, as the evidence was derived from observational studies. The evidence quality of studies on the effects of cold exposures (low temperature and cold spells) on cardiovascular disease–related mortality and morbidity was downgraded because of inconsistent results. All the *I*² >50% and 80% prediction intervals (PIs) included unity and were more than twice the random-effects meta-analysis confidence interval. Further, we upgraded the quality rating of the studies to moderate for the evident exposure-response gradients, except for the study on the effect of the cold spell on cardiovascular morbidity for its inconsistent dose response across studies (Supplementary Table S8).

## 4. Discussion

The present study aimed to clarify the effects of cold exposure (low temperature and cold spell) on cardiovascular disease–related health outcomes (mortality and morbidity) and explore the population’s susceptibilities to cold-induced cardiovascular diseases. We systematically reviewed 159 articles in the synthesis to strengthen the evidence on the increase in cardiovascular disease risk due to cold ambient exposures and to clarify the magnitude of cold impact. We provided new knowledge that the risk of cold exposure to cardiovascular diseases varies among climate zones. The meta-analysis indicated that following every 1°C decrease, cardiovascular-associated mortality increased by 1.6% and morbidity by 1.2%. A more substantial effect was observed in the morbidity of cardiac arrest and AAD, while the impact of cold exposure on hypertensive disease outcomes was not significant. Notably, cold spells significantly increased cardiovascular-related mortality and morbidity by 32.4% and 13.8%, respectively.

Our results update the findings of the previous studies and clarify the impact of cold exposure on cardiovascular outcomes with regard to both its direction and magnitude (11, 13, 14). Knowledge generated from previous studies was consistent in terms of direction, showing the positive association between cold exposure and cardiovascular disease outcomes, while its magnitude remained disputable. To better understand the extent of cold impact on cardiovascular disease, we conducted a wide-ranging search and analysis of current evidence with available information on daily temperature, location, and International Classification of Diseases-coded cause of death. Specifically, 80 studies exploring the association between low temperatures and cardiovascular disease were included in the meta-analysis. We found that with every 1°C drop in temperature, the RR of cardiovascular disease increased by 1.6%. We further conducted a series of sensitivity analyses by carrying out an elaborate stratification on included literature, considering the confounding factors. An analysis of different stratifications of the study also showed similar results for both direction and magnitude. These results suggested the robustness of our conclusion, which may be more in accord with the actual situation. Furthermore, we analyzed the impact of low temperatures on cardiovascular morbidity using the same method, which showed a 1.2% increase in cardiovascular mortality with every 1°C decrease. Notably, the impact of cold spells on cardiovascular disease was considerable, which increased mortality by 32.4% and morbidity by 13.8%. Our results provided the latest and unbiased evidence of the association between cold exposure and cardiovascular disease, which may help researchers better evaluate the impact of climate change.

The varied magnitude of cold impact suggests the existence of some crucial factors that could influence cold impact on cardiovascular health. Exploring these influential factors and the population’s susceptibility to cold-induced cardiovascular diseases is an important finding in our review. Here, we analyzed the cold effects in different climate conditions by stratifying the included articles using the Köppen–Geiger climate zones classification (20). As the results showed, the increased mortality caused by low-temperature exposure was more pronounced in a location with a higher mean daily temperature, such as the tropical climate zone (24.23°C; RR 1.023), Mediterranean climate zone (20.19°C; RR 1.024), and subtropical climate zone (19.78°C; RR1.032). In comparison, the cold effects were less pronounced in those with a lower mean daily temperature, such as the oceanic climate zone (10.38°C; RR 1.009), continental climate zone (14.60°C; RR 1.010), and subarctic climate zone (9.23°C; RR 1.009). Similar results have been found in clinical research worldwide (7, 29, 30). For example, Ebi and Mills reported that cold-related mortality increased significantly in regions with higher winter temperatures in the United Kingdom (29). Furthermore, Guo et al. found that the cold effects in southern China were more pronounced than in northern cities (7). Locations with higher mean temperatures tend to have higher optimal temperatures and to be intolerant to a fall in temperature, probably through physical adaptation (1). More importantly, social adaptation may play an even more critical role, as it is a known fact that the susceptible population, such as the elderly and patients with cardiovascular disease, should wear protective clothing and remain active in cold weather outdoors (29). However, The Eurowinter Group reported that in relatively warm countries, such a population often does not follow such practices because they do not feel the need (30). These findings suggest that excessive deaths in some instances could be avoided by way of the authorities taking several steps to promote subjective measures and public measures such as wind-proofing bus shelters. In addition, cold-related mortality is significantly higher in countries with lower-middle-income levels. The social capacity to adapt is also probably tied to economic development. People living in such countries may have less capacity to adapt to decreased temperatures, potentially exacerbating health inequalities across countries.

We further examined the cold impact on different kinds of cardiovascular diseases classified by the International Classification of Diseases-coded. Among them, cold exposure showed the most potent impact on the mortality of CHD and the morbidity of out-of-hospital cardiac arrest and AAD. In contrast, its role in hypertensive disease outcomes was not significant. Mechanically, the autonomic nervous system and humoral regulation system consist of a precise network to maintain blood pressure, which may not easily be disturbed by a change in ambient temperature. Moreover, our finding is consistent with that of a previous meta-analysis that explored the association between low temperatures and blood pressure. It was reported that a 1°C decrease in the mean daily outdoor temperature increased the systolic and diastolic blood pressure by 0.26 and 0.13 mmHg, respectively (12). These results suggested a possible correlation between decreased temperature and the incidence of hypertensive diseases, while the precise relationship remained largely unknown, which warrants future research. For example, research with a more detailed classification of the extent of temperature change and patients with underlying diseases is still needed. Recently, a meta-analysis that explored the effect of heat exposure on cardiovascular diseases reported a 2.8% and 17% increase in cardiovascular mortality followed by high temperatures and heat wave exposure, respectively (19). Coincidentally, both heat and cold exposure exercised the most substantial impact on cardiac arrest and minimum effect on hypertensive disease (19). Future exploration of the critical mechanism elicited by non-optimal temperatures may explain the results.

Cold temperatures could impact cardiovascular activity through many mechanisms. For example, cold exposure increases blood viscosity by elevating blood, platelet count, and red blood cell count in a few hours, which may increase the risk of ischemic heart disease and stroke (31–33). This could explain our study’s finding of increased risk of CHD and stroke after cold exposure. Furthermore, the present analysis suggested a high correlation between low temperatures and cardiac arrest morbidity, which may be explained by cold-induced autonomic nervous system disruption and inflammation–coagulation cascade activation (34–36). In addition, cold exposure was found to be associated with several risk factors for cardiovascular disease. It was reported that exposure to lower temperatures could be associated with a higher risk of metabolic derangement, including higher plasma glucose and more insulin resistance (37). Moreover, patients with diabetes were more prone to cold-related cardiovascular disease (38). Similarly, cold exposure impacted lipid metabolism disorder and influenza epidemics (39) and may induce more fat and alcohol intake.

The present synthesis showed a substantial interstudy heterogeneity. Considering the significant number of studies included in this meta-analysis, it is hard to avoid some inherent differences related to factors such as study design, meteorological variables, study population, and statistical mode. To analyze the source of heterogeneity, we carried out sensitivity analysis, subgroup analysis, and meta-regression, considering various covariant aspects such as temperature metrics, study design, study season, lag effects, air pollution adjustment, and cold spell intensity. However, all these analyses failed to reduce heterogeneity, indicating that other unmeasured factors still contribute to the cold effects on cardiovascular diseases as covariants, which still needs future research. In addition, a series of sensitivity analyses done by separating studies by various covariants and let-one-out analyses showed consistency in direction and magnitude, except for the impact of cold spells on cardiovascular morbidity. In this study, two (33%) studies could render the pooled effects of cold spells on cardiovascular disease morbidity insignificant when left out from the analysis, indicating the instability of the result. This inconsistency may be attributed to the small amount of evidence present in the synthesis. More importantly, there needs to be a clear definition and reference periods for cold spells, which may cause significant heterogeneity and various estimated effects (40, 41).

The main advantages of our estimates of risk attributed to cold exposure are as follows. To our knowledge, this review is the first to focus on the impact of cold weather on cardiovascular disease and to analyze the influential factors that cause differences in terms of cold impact. Notably, we found that cold exposure had the most powerful impact on CHD and AAD. Moreover, we identified the climate zone as an essential influential factor in terms of the impact of ambient cold exposure on cardiovascular disease. We also provided strong evidence of the impact of cold exposure on cardiovascular disease with regard to both its direction and magnitude by conducting a wide-ranging search and analysis of the current evidence and carrying out a series of sensitivity analyses that attest to the robustness of our findings. However, our study still has some limitations to be addressed. First, we unbiasedly included relative peer-reviewed literature. However, the available studies are far from conclusive, and the quality of several studies is a matter of concern. These may inevitably affect the quality of the pooled results, which suggests the requirement for rigor and better instruments in future research. Second, we found a high heterogeneity among included studies. Although we employed a series of subgroup analyses and meta-regression, the source of heterogeneity was not identified. Therefore, we used a random-effects model to pool individual estimates in studies quantitatively. However, considering the undetected source of heterogeneity and confounders, caution should be exercised when interpreting these pooled effect estimates. Third, we referred to the methodology of the previous meta-analysis and chose the lag RRs with the maximum risks (18, 19), which could lead to mistakes in the pooled results. For example, such extracted data could inevitably induce higher estimated RRs. Moreover, temperatures in the following lag days could affect the results in the form of an unadjusted confounder. However, which lag RR gives a true picture of cold impact remains largely unknown, and it is unlikely to make the best choice on the basis of the available evidence. This suggests the need for future research on the relationship between the lag days and the impact of temperature. Fourth, despite the great amount of literature included in the pooled estimates, there were still a small number of estimates in some subgroups such as the mortality and morbidity of hypertensive disease and health outcomes in lower-middle-income-level countries.

This systematic review and meta-analysis used the most up-to-date data assessment method and included 159 pieces of literature on cold exposure and cardiovascular disease outcomes. This study provided updated evidence that cold exposure (both low temperatures and cold spells) could elevate the risk of cardiovascular disease–related mortality and morbidity. Findings from this review also highlight that people living in warmer climate zones and lower-middle-income countries are more susceptible to cold-induced cardiovascular diseases. This study helps evaluate the current risk factors for cardiovascular diseases and provides important implications for future healthcare prevention strategies and resource allocation for high-risk populations. Given the increases in the frequency and intensity of consecutive cold climatic extremes, urgent attention is called for to devise more successful strategies to reduce risks.

## Data Availability

The original contributions presented in the study are included in the article/Supplementary Material, further inquiries can be directed to the corresponding authors.
