# Supplementary material for: A systematic review and meta-analysis of cold exposure and cardiovascular disease outcomes
Source: Front Cardiovasc Med. 2023 Mar 27;10:1084611. doi: 10.3389/fcvm.2023.1084611 (PMC10083291; doi:10.3389/fcvm.2023.1084611)
Supplement: Supplementary file 1 [file Datasheet1.pdf]

SUPPLEMENTARY MATERIAL

A systematic review and meta-analysis of cold exposure and cardiovascular health outcomes

Jie-Fu Fan†, Yu-Chen Xiao†, Lu-Yu Niu†, Wan-Yang Li, Wei-Zhong Wang & Yang-Kai Wang

Department of Marine Biomedicine and Polar Medicine, Naval Medical University (Second Military Medical University), Shanghai, China.

†These authors have contributed equally to this work.

Short title: Meta-analysis of cold exposure and cardiovascular diseases

Correspondence:

Prof. Yangkai Wang, Department of Marine Biomedicine and Polar Medicine, Naval Medical University, 880 Xiangyin Road, Shanghai 200433, China.

Email: wyangkai2005@smmu.edu.cn

And Prof. Weizhong Wang, Department of Marine Biomedicine and Polar Medicine, Naval Medical University, 880 Xiangyin Road, Shanghai 200433, China.

Email: weizhongwang@smmu.edu.cn

Table S1: Preferred Reporting Items for Systematic Reviews and Meta-Analyses (PRISMA) checklist[1].

Section and Topic	Item #	Checklist item	Location where item is reported
**TITLE**			
Title	1	Identify the report as a systematic review.	1
**ABSTRACT**			
Abstract	2	See the PRISMA 2020 for Abstracts checklist.	2
**INTRODUCTION**			
Rationale	3	Describe the rationale for the review in the context of existing knowledge.	4
Objectives	4	Provide an explicit statement of the objective(s) or question(s) the review addresses.	4
**METHODS**			
Eligibility criteria	5	Specify the inclusion and exclusion criteria for the review and how studies were grouped for the syntheses.	6
Information sources	6	Specify all databases, registers, websites, organisations, reference lists and other sources searched or consulted to identify studies. Specify the date when each source was last searched or consulted.	6
Search strategy	7	Present the full search strategies for all databases, registers and websites, including any filters and limits used.	S2
Selection process	8	Specify the methods used to decide whether a study met the inclusion criteria of the review, including how many reviewers screened each record and each report retrieved, whether they worked independently, and if applicable, details of automation tools used in the process.	6
Data collection process	9	Specify the methods used to collect data from reports, including how many reviewers collected data from each report, whether they worked independently, any processes for obtaining or confirming data from study investigators, and if applicable, details of automation tools used in the process.	7
Data items	10a	List and define all outcomes for which data were sought. Specify whether all results that were compatible with each outcome domain in each study were sought (e.g. for all measures, time points, analyses), and if not, the methods used to decide which results to collect.	7
	10b	List and define all other variables for which data were sought (e.g. participant and intervention characteristics, funding sources). Describe any assumptions made about any missing or unclear information.	7
Study risk of bias assessment	11	Specify the methods used to assess risk of bias in the included studies, including details of the tool(s) used, how many reviewers assessed each study and whether they worked independently, and if applicable, details of automation tools used in the process.	8
Effect measures	12	Specify for each outcome the effect measure(s) (e.g. risk ratio, mean difference) used in the synthesis or presentation of results.	7
Synthesis methods	13a	Describe the processes used to decide which studies were eligible for each synthesis (e.g. tabulating the study intervention characteristics and comparing against the planned groups for each synthesis (item #5)).	7
	13b	Describe any methods required to prepare the data for presentation or synthesis, such as handling of missing summary statistics, or data conversions.	7
	13c	Describe any methods used to tabulate or visually display results of individual studies and syntheses.	7
	13d	Describe any methods used to synthesize results and provide a rationale for the choice(s). If meta-analysis was performed, describe the model(s), method(s) to identify the presence and extent of statistical heterogeneity, and software package(s) used.	8
	13e	Describe any methods used to explore possible causes of heterogeneity among study results (e.g. subgroup analysis, meta-regression).	8
	13f	Describe any sensitivity analyses conducted to assess robustness of the synthesized results.	8
Reporting bias assessment	14	Describe any methods used to assess risk of bias due to missing results in a synthesis (arising from reporting biases).	8, S6-9
Certainty	15	Describe any methods used to assess certainty (or confidence) in the body of evidence for an outcome.	8, S9

Section and Topic	Item #	Checklist item	Location where item is reported
assessment			
**RESULTS**			
Study selection	16a	Describe the results of the search and selection process, from the number of records identified in the search to the number of studies included in the review, ideally using a flow diagram.	8-9
	16b	Cite studies that might appear to meet the inclusion criteria, but which were excluded, and explain why they were excluded.	9
Study characteristics	17	Cite each included study and present its characteristics.	S4-5
Risk of bias in studies	18	Present assessments of risk of bias for each included study.	12,S16
Results of individual studies	19	For all outcomes, present, for each study: (a) summary statistics for each group (where appropriate) and (b) an effect estimate and its precision (e.g. confidence/credible interval), ideally using structured tables or plots.	9,S10-13
Results of syntheses	20a	For each synthesis, briefly summarise the characteristics and risk of bias among contributing studies.	9,11
	20b	Present results of all statistical syntheses conducted. If meta-analysis was done, present for each the summary estimate and its precision (e.g. confidence/credible interval) and measures of statistical heterogeneity. If comparing groups, describe the direction of the effect.	9-11,S15-16
	20c	Present results of all investigations of possible causes of heterogeneity among study results.	11,S15-16
	20d	Present results of all sensitivity analyses conducted to assess the robustness of the synthesized results.	11,S14-15
Reporting biases	21	Present assessments of risk of bias due to missing results (arising from reporting biases) for each synthesis assessed.	S18-22
Certainty of evidence	22	Present assessments of certainty (or confidence) in the body of evidence for each outcome assessed.	12,17
**DISCUSSION**			
Discussion	23a	Provide a general interpretation of the results in the context of other evidence.	13-14
	23b	Discuss any limitations of the evidence included in the review.	17
	23c	Discuss any limitations of the review processes used.	17
	23d	Discuss implications of the results for practice, policy, and future research.	17
**OTHER INFORMATION**			
Registration and protocol	24a	Provide registration information for the review, including register name and registration number, or state that the review was not registered.	6
	24b	Indicate where the review protocol can be accessed, or state that a protocol was not prepared.	6
	24c	Describe and explain any amendments to information provided at registration or in the protocol.	6
Support	25	Describe sources of financial or non-financial support for the review, and the role of the funders or sponsors in the review.	18
Competing interests	26	Declare any competing interests of review authors.	18
Availability of data, code and other materials	27	Report which of the following are publicly available and where they can be found: template data collection forms; data extracted from included studies; data used for all analyses; analytic code; any other materials used in the review.	6

Table S2: Search strategy: databases, terms, filters and number of articles for review.

**Database**	**Strategy**	**Number of hits**	**Number imported into Endnote**
**PubMed**	#1 weather [MH] OR climate change [MH] OR temperature [TW] OR season [TW] OR cold temperature [TW] OR cold season [TW] OR cold spell [TW] OR cold weather	228,591	

**Filters:**<br>**English, Human,**<br>**Full text**	[TW]<br><br>#2 cardiovascular diseases [MH] OR heart diseases [TW] OR vascular diseases [TW] OR hypertensive disease [TW] OR myocardial infarction [TW] OR stroke [TW] OR heart failure [TW] OR arrhythmia [TW] OR cardiac arrest [TW] OR rheumatic heart disease [TW] OR thrombosis [TW] OR thrombotic disease [TW] OR pulmonary heart disease [TW] OR peripheral artery disease [TW] OR aortic aneurysm [TW] OR aortic dissection [TW]<br><br>#3 epidemiology [MH] OR mortality [TW] OR morbidity [TW] OR incidence [TW] OR prevalence [TW] OR outbreak [TW] OR surveillance [TW] OR occurrence [TW] OR hospital [TW] OR death [TW]<br><br>#1 AND #2 AND #3	 <br>2,915,117<br><br>2,963,121	9,675
**Cochrane**<br><br>**Filters:**<br>**In trails**	#1 ("climate change"):ti,ab,kw OR (temperature):ti,ab,kw OR (season):ti,ab,kw OR ("cold spell"):ti,ab,kw OR ("cold weather"):ti,ab,kw (Word variations have been searched)<br>#2 MeSH descriptor: [Weather] explode all trees<br>#3("heart diseases"):ti,ab,kw OR ("vascular diseases"):ti,ab,kw OR ("hypertensive disease"):ti,ab,kw OR ("myocardial infarction"):ti,ab,kw OR (stroke):ti,ab,kw<br>#4 ("heart failure"):ti,ab,kw OR (arrhythmia):ti,ab,kw OR ("cardiac arrest"):ti,ab,kw<br>#5("rheumatic heart disease"):ti,ab,kw OR ("thrombotic disease"):ti,ab,kw OR ("pulmonary heart disease"):ti,ab,kw OR ("aortic aneurysm and dissection"):ti,ab,kw OR ("peripheral artery disease"):ti,ab,kw<br>#6 MeSH descriptor: [Cardiovascular Diseases] explode all trees<br>#7 (mortality):ti,ab,kw OR (morbidity):ti,ab,kw OR (incidence):ti,ab,kw AND (prevalence):ti,ab,kw AND (outbreak):ti,ab,kw<br>#8 (surveillance):ti,ab,kw OR (occurrence):ti,ab,kw OR (hospital):ti,ab,kw AND (death):ti,ab,kw<br>#9 MeSH descriptor: [Epidemiology] explode all trees<br>#10 #1 OR #2<br>#11 #3 OR #4 OR #5 OR #6<br>#12 #7 OR #8 OR #9<br>#13 #10 AND #11 AND#12	35,192<br>6,125<br>110,237<br>46,428<br>15,977<br>118,039<br>128,815<br>60,131<br>42<br>36559<br>445,827<br>171,706	1,733
**Scopus**<br><br>**Filters:**<br>**English,**<br>**Article,**	#1 (ALL ("climate change") OR TITLE-ABS-KEY (weather) OR TITLE-ABS-KEY ("low temperature") OR TITLE-ABS-KEY (season) OR TITLE-ABS-KEY ("cold temperature") OR TITLE-ABS-KEY ("cold spell") OR TITLE-ABS-KEY ("cold weather") OR TITLE-ABS-KEY ("cold season"))<br><br>#2 (ALL ("cardiovascular diseases ") OR TITLE-ABS-KEY ("hypertensive disease") OR TITLE-ABS-KEY ("heart diseases") OR TITLE-ABS-KEY ("vascular diseases") OR TITLE-ABS-KEY (stroke) OR TITLE-ABS-KEY ("myocardial infarction") OR TITLE-ABS-KEY ("heart failure") OR TITLE-ABS-KEY (arrhythmia) OR	2,374,448<br><br>3,016,495	

	TITLE-ABS-KEY ("cardiac arrest") OR TITLE-ABS-KEY (“rheumatic heart disease”) OR TITLE-ABS-KEY (“thrombotic disease”) OR TITLE-ABS-KEY (thrombosis) OR TITLE-ABS-KEY (“pulmonary heart disease") OR TITLE-ABS-KEY ("aortic aneurysm”) OR TITLE-ABS-KEY ("aortic dissection”) OR TITLE-ABS-KEY (“peripheral artery disease”))<br><br>#3 (ALL (epidemiology) OR TITLE-ABS-KEY (mortality) OR TITLE-ABS-KEY (morbidity) OR TITLE-ABS-KEY (prevalence) OR TITLE-ABS-KEY (incidence) OR TITLE-ABS-KEY ("hospital admission") OR TITLE-ABS-KEY (occurrence))<br>#1 AND #2 AND #3	7,885,815	10,316
**Total results**			21,724

All the included articles[2-160].

Table S3: Rating tool of risk of bias assessment of included study, modified according to the OHAT[161,162].

	**Risk of Bias Questions**	**Answer**
**Key Criteria**		
**Exposure assessment**	Includes measurement error or measurement limitations.<br>List of major considerations:<br>1) more than one monitoring station per a large geographical area<br>2) daily ambient temperature measurements were available	-LOW: There is high confidence that the exposure to ambient temperature is the true average population exposure (e.g. using gridded temperature data-interpolated or population weighted temperature).<br><br>-PROBABLY LOW: There is indirect evidence that suggests low risk of bias, or one of the three listed considerations is not applied (e.g. averaged temperatures from several weather stations).<br><br>-PROBABLY HIGH: There is insufficient information to permit a judgment of high risk of bias, but there is indirect evidence that suggests high risk of bias. Additionally, two out of the three listed considerations are not applied (e.g. use of single weather station).<br><br>-HIGH: There is direct evidence of high risk of misclassification bias, or all three of the listed considerations are not applied.
**Outcome assessment**	Includes blinding, systematic errors, or not comparable outcome measurement<br>across exposure groups.<br>List of major considerations:<br>1) outcome measurements were not influenced by knowledge of the exposure (data were obtained from different databases)<br>2) validity of disease classification methods (ICD coding)	-LOW: mortality and morbidity cause classified based on diagnosis standard criteria (International Classification System – ICD code) and provided by a National or Regional Database.<br><br>-PROBABLY LOW: outcome was assessed based on diagnosis standard criteria (ICD) and collected by researcher, but did not specify the data source.<br><br>-PROBABLY HIGH: outcome was not assessed based on standard diagnosis criteria. Additionally, there is evidence that suggests the existence of misclassification bias.<br><br>-HIGH: Outcome was assessed based on self-reports (parents, family) and data

		collected by the researcher. Additionally, there is evidence that suggests the high risk of misclassification bias.
**Confounding bias**	List of major considerations:<br>1) study appropriately accounted for all important well studied potential confounders (seasonality, time trends, day of week, relative humidity).4<br>2) did the authors use an appropriate analysis method or study design that controlled for confounding domains?	-LOW: study accounted for all important confounders which were measured consistently.<br><br>-PROBABLY LOW: study accounted for most of confounders AND is not expected to introduce bias.<br><br>-PROBABLY HIGH: study accounted for some but not all confounders AND is expected to introduce bias<br><br>-HIGH: study did not account for potential confounders OR were inappropriately measured
**Other Criteria**		
**Selection/recruitment bias**	List of major considerations:<br>1) does the selection of participants into the study was done in a manner that might introduce bias in the study?<br>(e.g. study only certain days, and not all days, seasons were included)	-LOW: The descriptions of the studied population were sufficiently detailed to support the assertion that risk of selection effects was minimal (e.g., all relevant mortality and morbidity outcomes in the study setting were reported and included in the study, participants in all exposure levels and with all outcomes had equal opportunity to be included in the study).<br><br>-PROBABLY LOW: There is insufficient information about population selection to permit a judgment of low risk of bias, but there is indirect evidence that suggests low risk of bias (e.g.,<br>participants in all exposure levels may not have equal opportunity to be in the study).<br><br>-PROBABLY HIGH: There is insufficient information about population selection to permit a judgment of high risk of bias, but there is indirect evidence that suggests high risk of bias<br>(e.g., participants in all exposure levels did not have equal opportunity to be in the study; but not to the extent that seriously bias the effect estimates).<br><br>- HIGH: There were indications from descriptions of the studied population of high risk of bias (study only included designated high-risk participants, and participants in all exposure levels did not have equal opportunity to be in the study, to the extent that effect estimates were seriously biased).
**Incomplete outcome data**	List of major considerations:<br>1) missing data of outcome measures?<br>2) missing data of exposures?	-LOW: no missing outcome data or missing data are unrelated to true outcome.<br><br>-PROBABLY LOW: there was insufficient information about incomplete data to judge for low risk, but indirect evidence that suggests low risk of bias (e.g., <10% missing data, or missing data related to outcome or exposure data imputed using appropriate method).

		-PROBABLY HIGH: there was insufficient information about incomplete data to judge for high risk, but indirect evidence that suggests high risk bias (e.g., ≥10% missing data without<br>imputed using appropriate method, while rational for attrition explained in the manuscript with possible methods have been used to properly account for it).<br><br>-HIGH: missing outcome data are related to true outcome (e.g., substantial missing exposure data (≥10%), rationale for missing data not explained in the manuscript).
**Selective reporting**	List of major considerations:<br>1) do the authors report a prior primary and secondary study aims?<br>2) study reports data analysis over a complete or original database, with no selective reporting of outcomes or analysis	-LOW: all of the studies pre-specified outcomes and findings are reported (i.e., effect estimates presented for all hypothesis tested as per aims)<br><br>-PROBABLY LOW: there was insufficient information about selective outcome to judge for low risk, but indirect evidence that suggests study was free of selective report (i.e., effect<br>estimates presented for less than all hypotheses tested as per aims; but evidence suggests that effect estimates unlikely to be seriously biased).<br><br>-PROBABLY HIGH: there was insufficient information about selective reporting to judge for high risk, but indirect evidence suggests that study was not free of selective reporting<br><br>-HIGH: not all pre-specified outcomes and findings were reported (i.e., effect estimates presented for less than all hypotheses tested as per aims, and with direct evidence suggest that effect estimates likely to be seriously biased)
**Conflict of interest**	Potential source of bias in reporting through source of funding	-LOW: the study did not receive funding from an entity with financial interest in the outcome of study (e.g. funding source is limited to government or academic grants, authors make a<br>claim denying conflicts of interest)<br><br>-PROBABLY LOW: there is insufficient information to judge for low risk, but indirect evidence suggests study was free of financial interest<br><br>-PROBABLY HIGH: there is insufficient information to judge for high risk, but indirect evidence suggests study was not free of financial interest<br><br>-HIGH: study received support from an entity with financial interest in the outcome of study (e.g., authors/staff from study was employee or otherwise affiliated with an entity with<br>financial interest in the study outcome, authors claim a conflict of interest)
**Other sources of bias**	Bias due to other problems not covered elsewhere	-LOW: No other sources of bias<br><br>-PROBABLY LOW: there is insufficient information to judge for low risk, but indirect evidence suggests study was free of other problems

		-PROBABLY HIGH: there is insufficient information to judge for high risk, but indirect evidence suggests study was not free of other problems<br><br>-HIGH: at least one important risk of bias (e.g., selective reporting of subgroups, a potential source of bias related to the specific study design used, study has been claimed to have been<br>fraudulent)

Table S4: Judgement of overall risk of bias rating.

**Overall Rating**	**Combinations (three key components of exposure assessment, outcome assessment, and confounding bias)**
**High (H)**	High + High + (High / Probably high / Probably low / Low)<br>High + Probably high + Probably high
**Probably high (PH)**	High + Probably high + (Probably low / Low)<br>Probably high + Probably high + (Probably high / Probably low / Low)
**Probably low (PL)**	Probably high + (Probably low / Low) + (Probably low / Low)<br>Probably low + Probably low + Low
**Low (L)**	Probably low + Low + Low<br>Low + Low + Low

Table S5: Rating tool of evaluation of quality and strength of the body of evidence. According to Johnson et al[163].

**Evaluation factors**	**Summary of criteria**
**Downgrading factors**	
**Risk of bias**	Study limitations include a substantial risk of bias across the body of evidence. Risk of bias was assessed by sensitivity analyses, excluding studies rated “high” and/or “probably high” risk of bias. The quality of body of evidence was downgraded if there was substantial difference between values of sensitivity analysis.
**Indirectness**	Evidence was not directly comparable to the primary objective of interest i.e., participants, exposure, comparisons, outcome (PECO).
**Inconsistency**	Estimates of effect in similar populations were widely different (significantly high heterogeneity *I 2* or variability in results). In addition, the evidence was downgraded if the 80% precision interval included unity and was more than twice the random effects meta-analysis confidence interval.
**Imprecision**	Studies included few participants and small sample sizes (wide confidence interval as judged by reviewers).
**Publication bias**	Studies were missing from body of evidence, resulting in an over- or underestimate of true effects from exposure. The evidence of publication bias was inspected visually in the funnel plots and egger’s test. The *Trim and Fill* procedure was used to estimate potentially missing studies.
**Upgrading factors**	
**Large magnitude of effect**	The rating was upgraded if modeling suggested that confounding alone was unlikely to explain associations that were judged to be of large magnitude.
**Dose response**	Upgraded if consistent relationship between dose and response in one or multiple studies, and/or the dose response across studies.
**Confounding minimizes effect**	Upgraded if the consideration of all plausible residual confounders or biases would underestimate the effect or suggest a spurious effect when results show no effect.

Figure S1: Forest plot of each study investigating the association between low temperature and cardiovascular disease mortality, with every 1°C decrease in temperature.

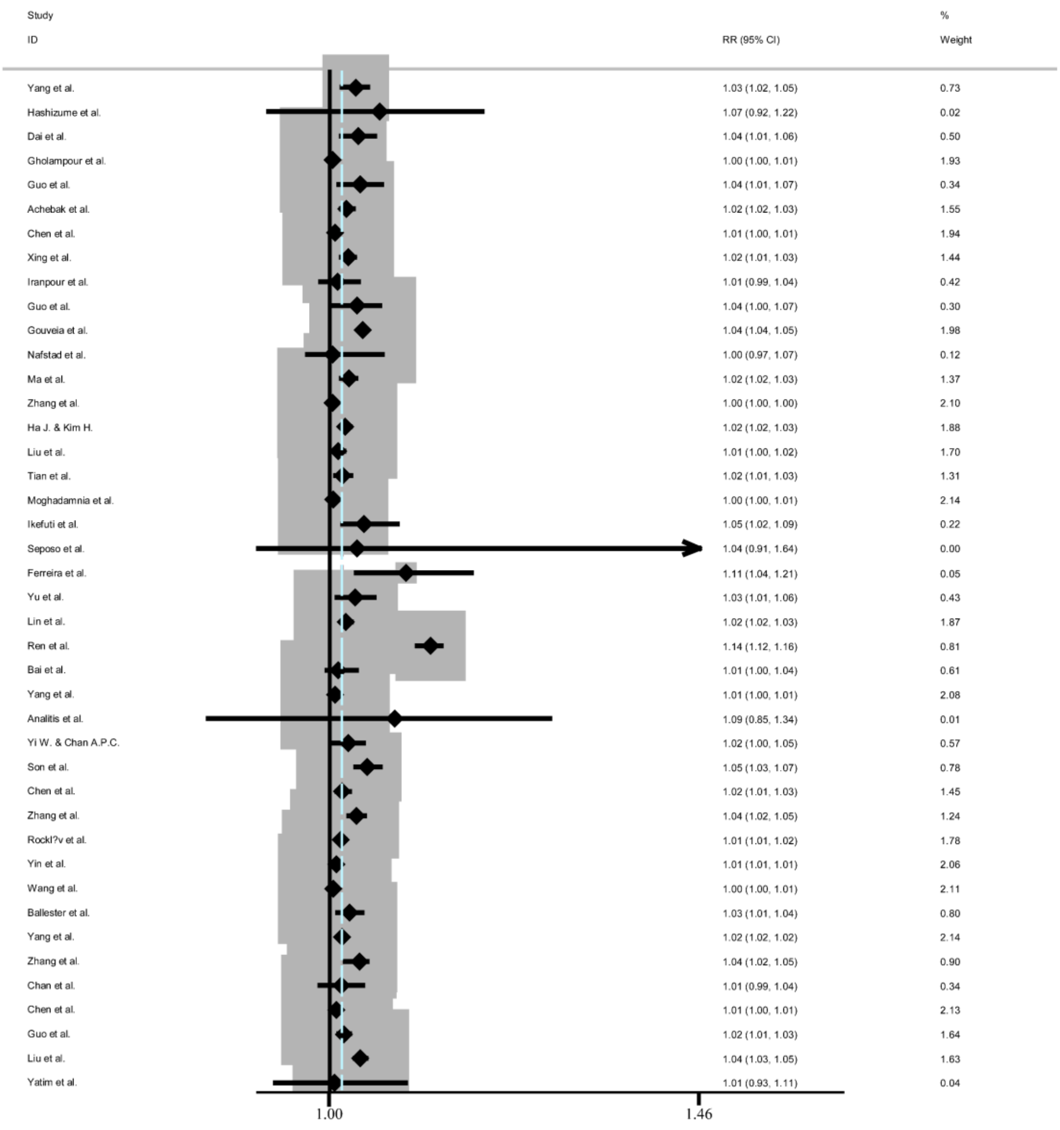

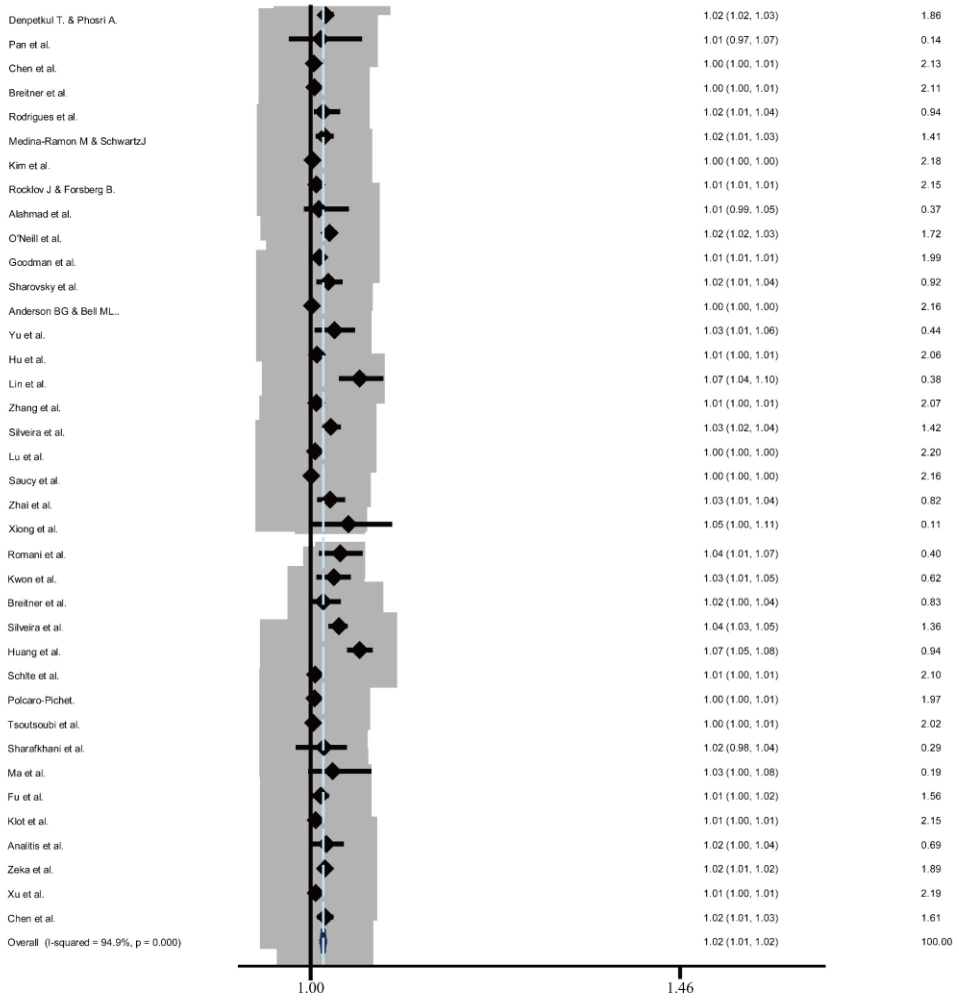

Denpetkul T. & Phosri A. 1.02 (1.02, 1.03) 1.86
Pan et al. 1.01 (0.97, 1.07) 0.14
Chen et al. 1.00 (1.00, 1.01) 2.13
Breitner et al. 1.00 (1.00, 1.01) 2.11
Rodrigues et al. 1.02 (1.01, 1.04) 0.94
Medina-Ramon M & SchwartzJ 1.02 (1.01, 1.03) 1.41
Kim et al. 1.00 (1.00, 1.00) 2.18
Rocklov J & Forsberg B. 1.01 (1.01, 1.01) 2.15
Alahmad et al. 1.01 (0.99, 1.05) 0.37
O'Neill et al. 1.02 (1.02, 1.03) 1.72
Goodman et al. 1.01 (1.01, 1.01) 1.99
Sharovsky et al. 1.02 (1.01, 1.04) 0.92
Anderson BG & Bell ML.. 1.00 (1.00, 1.00) 2.16
Yu et al. 1.03 (1.01, 1.06) 0.44
Hu et al. 1.01 (1.00, 1.01) 2.06
Lin et al. 1.07 (1.04, 1.10) 0.38
Zhang et al. 1.01 (1.00, 1.01) 2.07
Silveira et al. 1.03 (1.02, 1.04) 1.42
Lu et al. 1.00 (1.00, 1.00) 2.20
Saucy et al. 1.00 (1.00, 1.00) 2.16
Zhai et al. 1.03 (1.01, 1.04) 0.82
Xiong et al. 1.05 (1.00, 1.11) 0.11
Romani et al. 1.04 (1.01, 1.07) 0.40
Kwon et al. 1.03 (1.01, 1.05) 0.62
Breitner et al. 1.02 (1.00, 1.04) 0.83
Silveira et al. 1.04 (1.03, 1.05) 1.36
Huang et al. 1.07 (1.05, 1.08) 0.94
Schlte et al. 1.01 (1.00, 1.01) 2.10
Polcaro-Pichet. 1.00 (1.00, 1.01) 1.97
Tsoutsoubi et al. 1.00 (1.00, 1.01) 2.02
Sharafkhani et al. 1.02 (0.98, 1.04) 0.29
Ma et al. 1.03 (1.00, 1.08) 0.19
Fu et al. 1.01 (1.00, 1.02) 1.56
Klot et al. 1.01 (1.00, 1.01) 2.15
Analitis et al. 1.02 (1.00, 1.04) 0.69
Zeka et al. 1.02 (1.01, 1.02) 1.89
Xu et al. 1.01 (1.00, 1.01) 2.19
Chen et al. 1.02 (1.01, 1.03) 1.61
Overall (I-squared = 94.9%, p = 0.000) 1.02 (1.01, 1.02) 100.00
1.00
1.46

Figure S2: Forest plot of each study investigating the association between low temperature and cardiovascular disease morbidity, with every 1°C decrease in temperature.

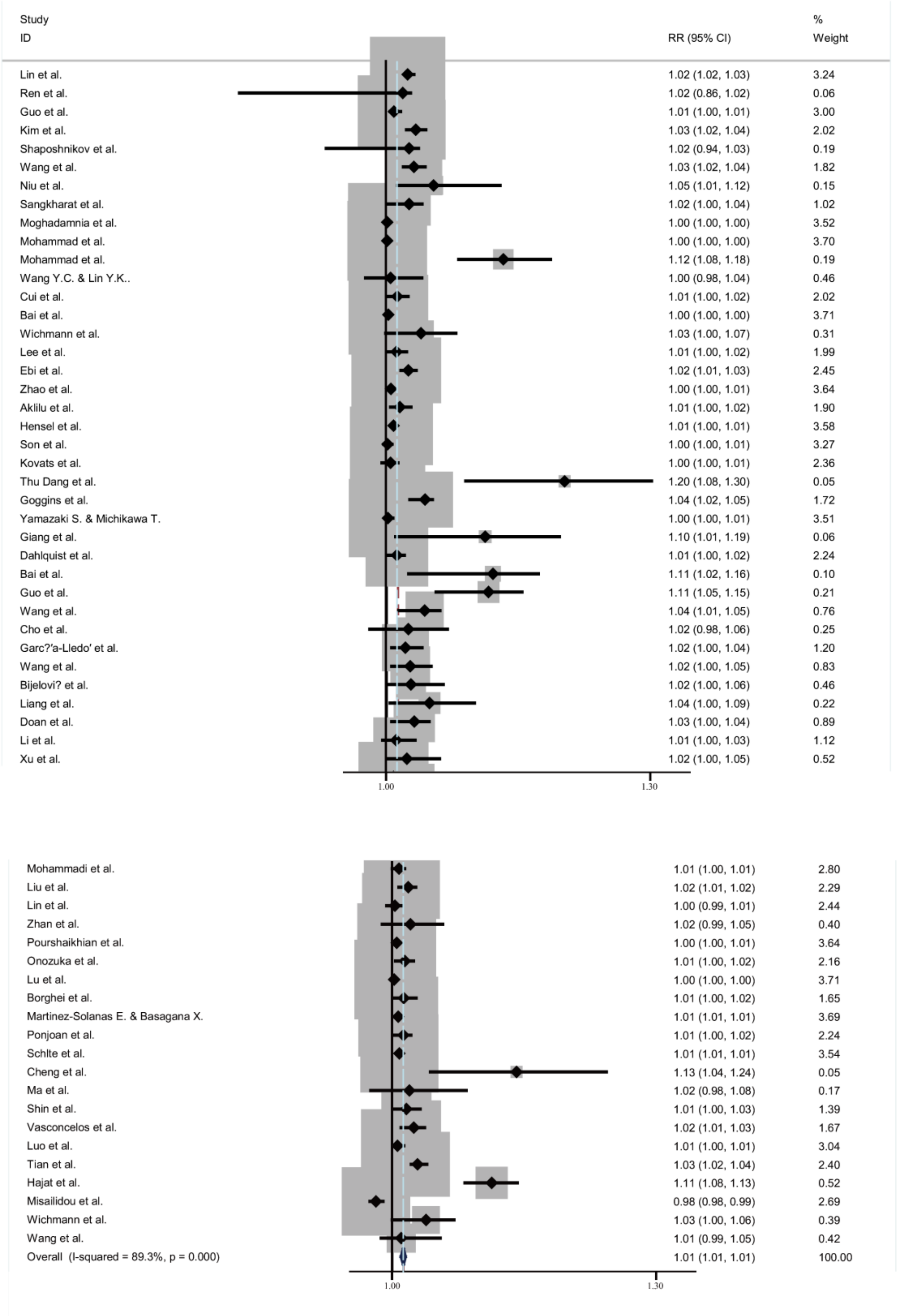

Figure S3: Forest plot of each study investigating the association between cold spells and cardiovascular disease mortality.

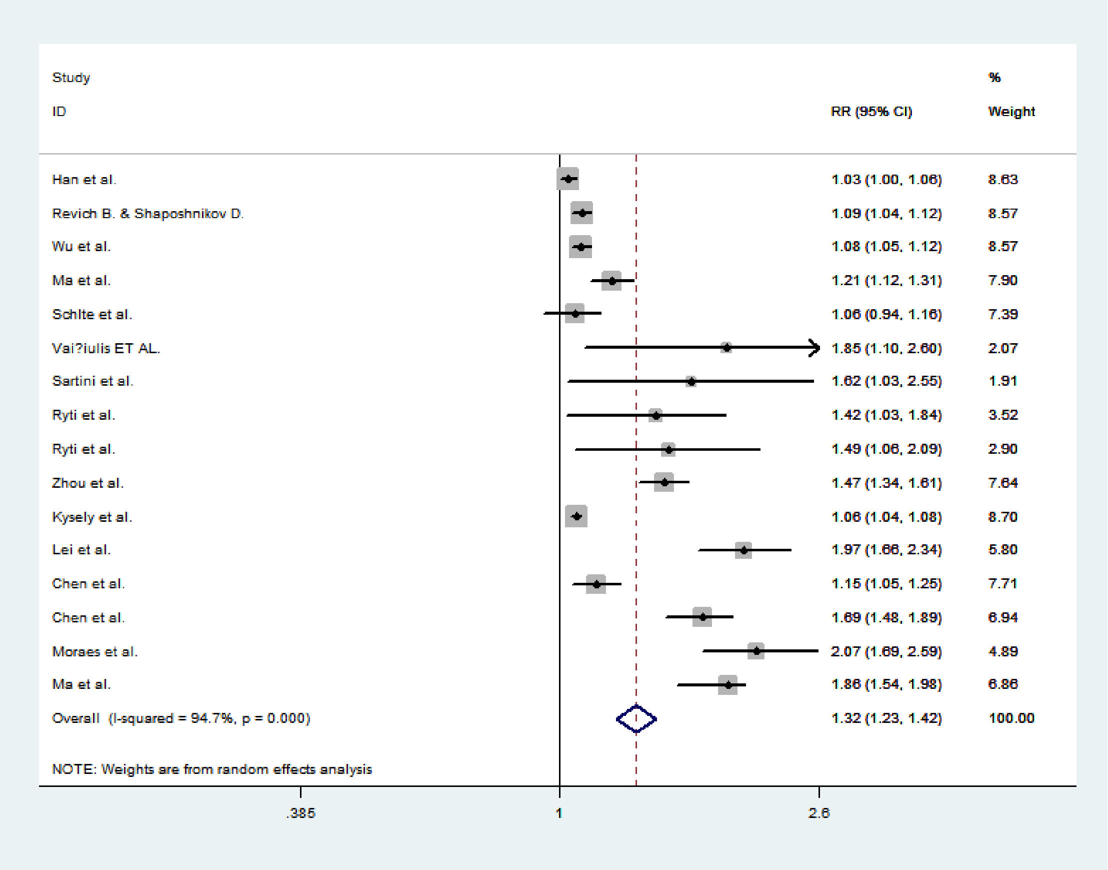

Figure S4: Forest plot of each study investigating the association between cold spells and cardiovascular disease morbidity.

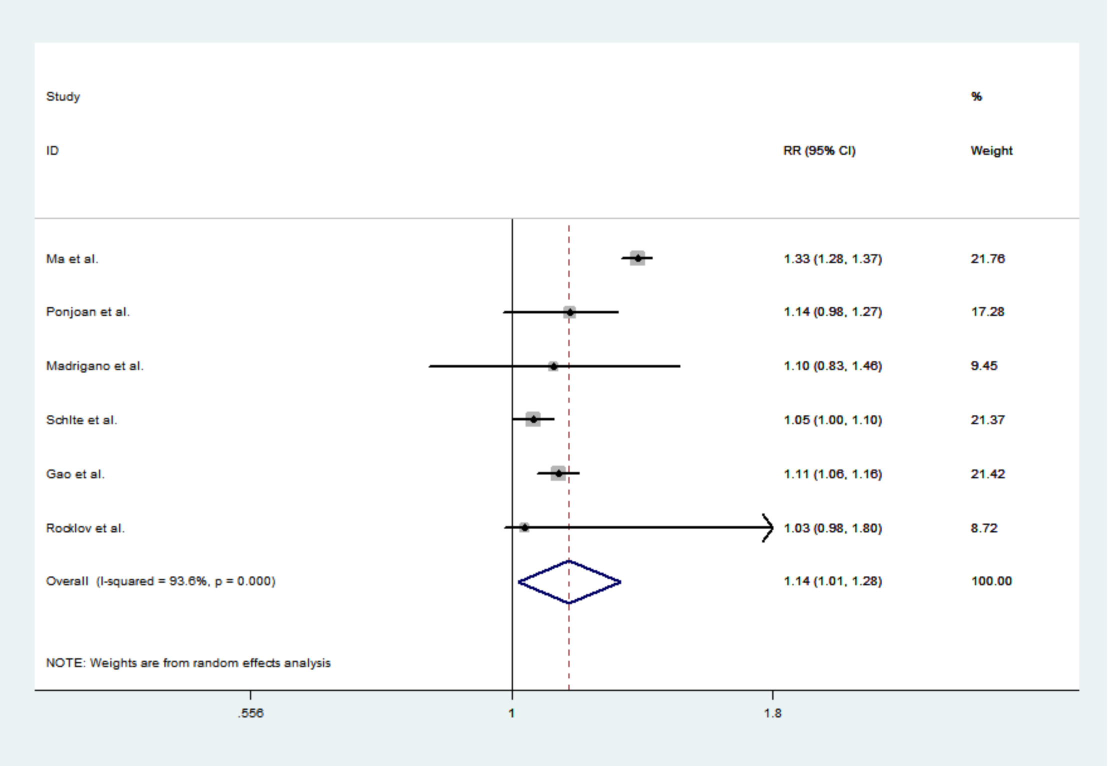

Table S6: Meta-regression model investigating the predictors of log pooled effect sizes for the associations between low temperatures and cardiovascular mortality and morbidity.

**Regressors**	**Mortality, β (95% CIs,)**
**Climate zone (ref = Group A-Tropical)**	
Group B-Dry	1.005(0.956-1.057)
Group C-Mediterranean	1.011 (0.967-1.056)
Group C-Oceanic	1.021 (0.981-1.063)
Group C-Subtropical	1.013 (0.989-1.038)
Group D-Continental	1.014 (0.987-1.042)
Group E-Subarctic	1.002 (0.954-1.053)
**National income level (ref = high income)**	
Upper-middle income	0.993 (0.974-1.014)
Lower-middle income	1.124 (1.035-1.221)*
Annual mean temperature	1.000 (0.998-1.002)
Latitude	1.000 (1.000-1.000)
Longitude	1.000 (1.000-1.000)
Constant	1.013 (0.944-1.086)
**Regressors**	**Morbidity, β (95% CIs,)**
**Climate zone (ref = Group E-Subarctic)**	
Group B-Dry	1.005 (0.956-1.057)
Group C-Mediterranean	1.020 (0.976-1.067)
Group C-Oceanic	1.021 (0.981-1.063)
Group C-Subtropical	1.013(0.989-1.038)
Group D-Continental	1.014 (0.987-1.042)
**National income level (ref = high income)**	
Upper-middle income	0.993 (0.974-1.014)
Lower-middle income	1.124 (1.035-1.221)*
Annual mean temperature	1.000 (0.998-1.002)
Latitude	1.000 (1.000-1.000)
Longitude	1.000 (1.000-1.000)
Constant	1.012 (0.944-1.086)

* , statistically significant (p-value<0·05). 95% CI, 95% Confidence interval.

Table S7: Meta-regression testing for differences between subgroups for the associations between cold spells and cardiovascular mortality and morbidity.

**Subgroups**	**Mortality (p-value)**	**Morbidity (p-value)**
**National income (ref=high income)**		
Upper-middle-income	0.49	0.14
**Climate zone (ref=Group E-subarctic)**		
Group C-Ocean	0.99	..
Group C-Subtropical	0.99	0.25

Group C-Continal	0.97	0.89
**Cold spell intensity (ref=high intensity)**		
Middle intensity	0.99	0.45
Low intensity	0.89	..

Figure S5: Risk of bias assessment of individual studies.

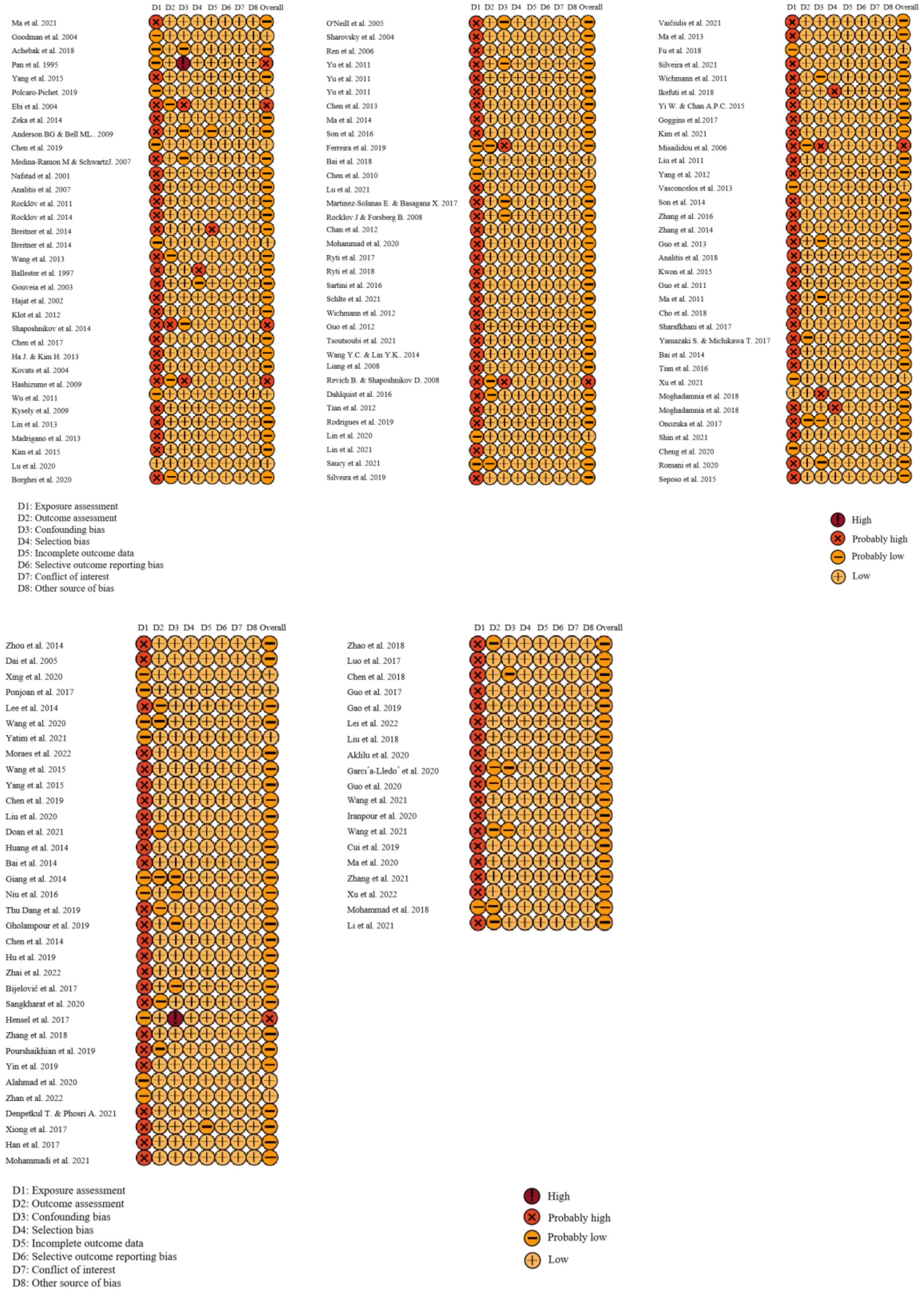

Figure S6: Weighted bar plots of the risk of bias assessment of included studies on low temperatures and cardiovascular disease mortality (A), low temperatures and cardiovascular disease morbidity (B), cold spells and cardiovascular mortality (C), and cold spells and cardiovascular morbidity (D).

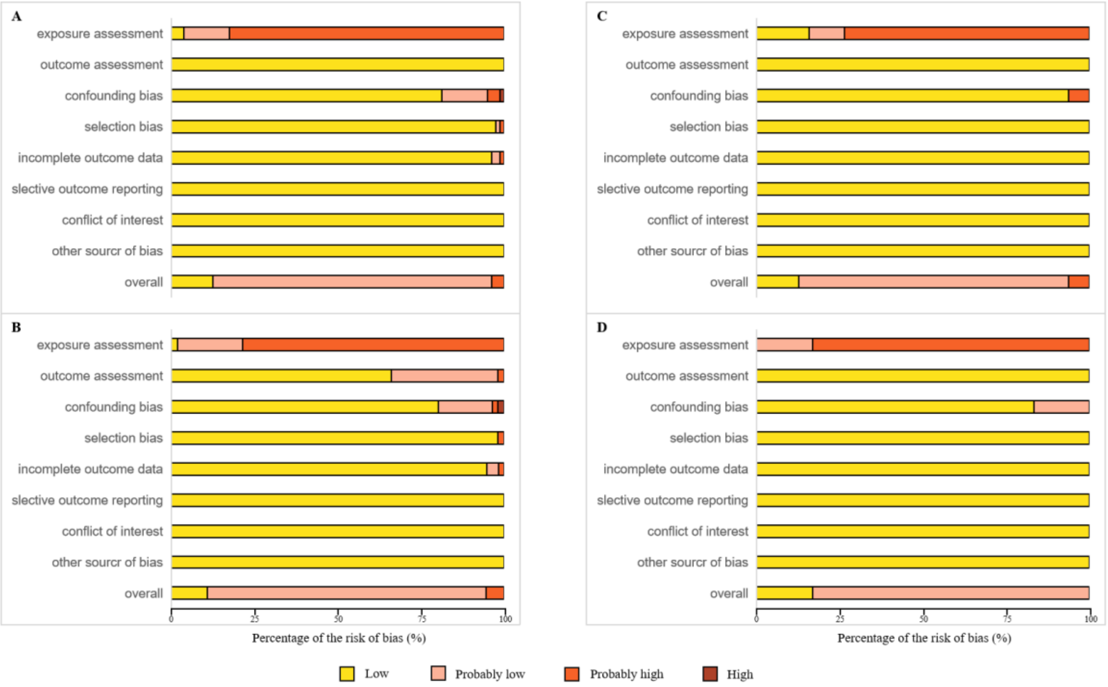

Table S8: Rating of quality and strength of the body of evidence of influence of low temperature and cold spells on cardiovascular mortality and morbidity.

	**Low temperature studies**		**Cold spell studies**	
	**Mortality**	**morbidity**	**Mortality**	**morbidity**
**Quality of evidence assessment**				
**Downgrade**				
	**Rating**	**Rating**	**Rating**	**Rating**
**-Risk of bias across studies**	(0)	(0)	(0)	(0)
**-Indirectness**	(0)	(0)	(0)	(0)
**-Inconsistency**	(-1)	(-1)	(-1)	(-1)
**-Imprecision**	(0)	(0)	(0)	(0)
**-Publication Bias**	(0)	(0)	(0)	(0)
**Upgrade**				
**-Large magnitude of effect**	(0)	(0)	(0)	(0)

**-Dose response**	(+1)	(+1)	(+1)	(0)
**-Confounding minimizes the effect**	(0)	(0)	(0)	(0)
**Summary of the quality assessment**				
**-Overall quality of evidence (initial rating is "moderate")**	Moderate	Moderate	Moderate	Low

Figure S7: Funnel plots to explore publication bias for studies investigating the association between low temperature and cardiovascular mortality.

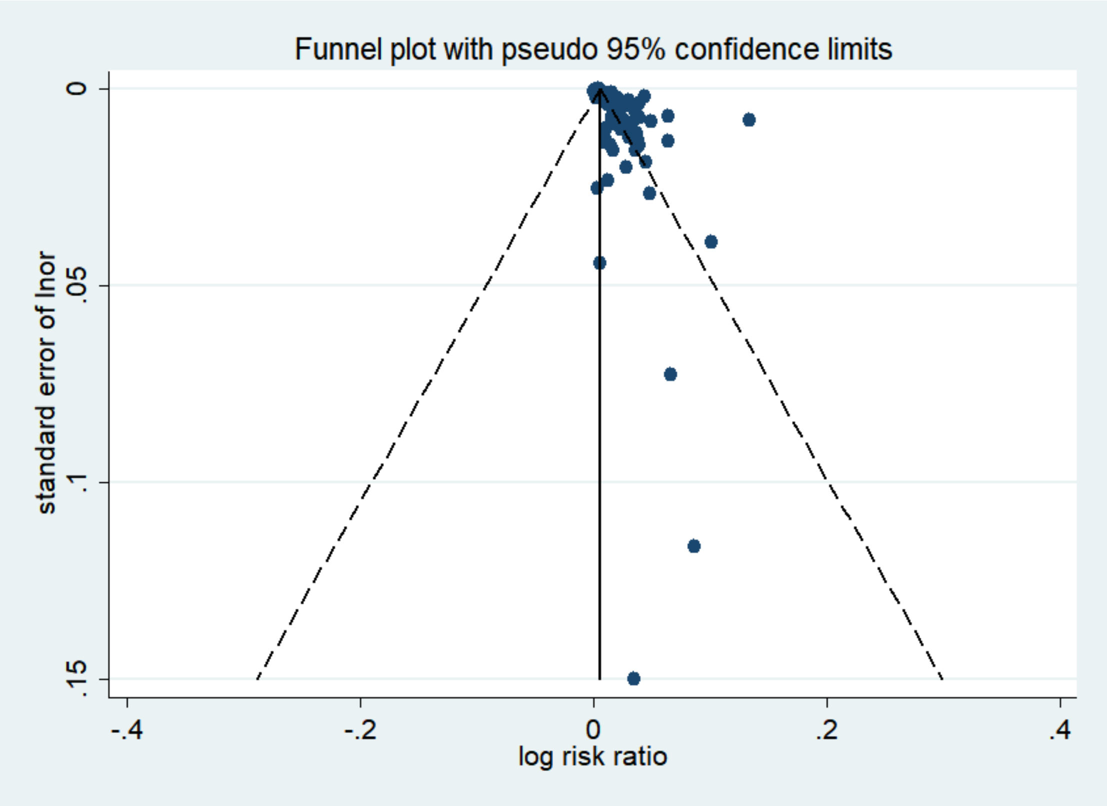

Figure S8: Funnel plots to explore publication bias for studies investigating the association between low temperature and cardiovascular morbidity.

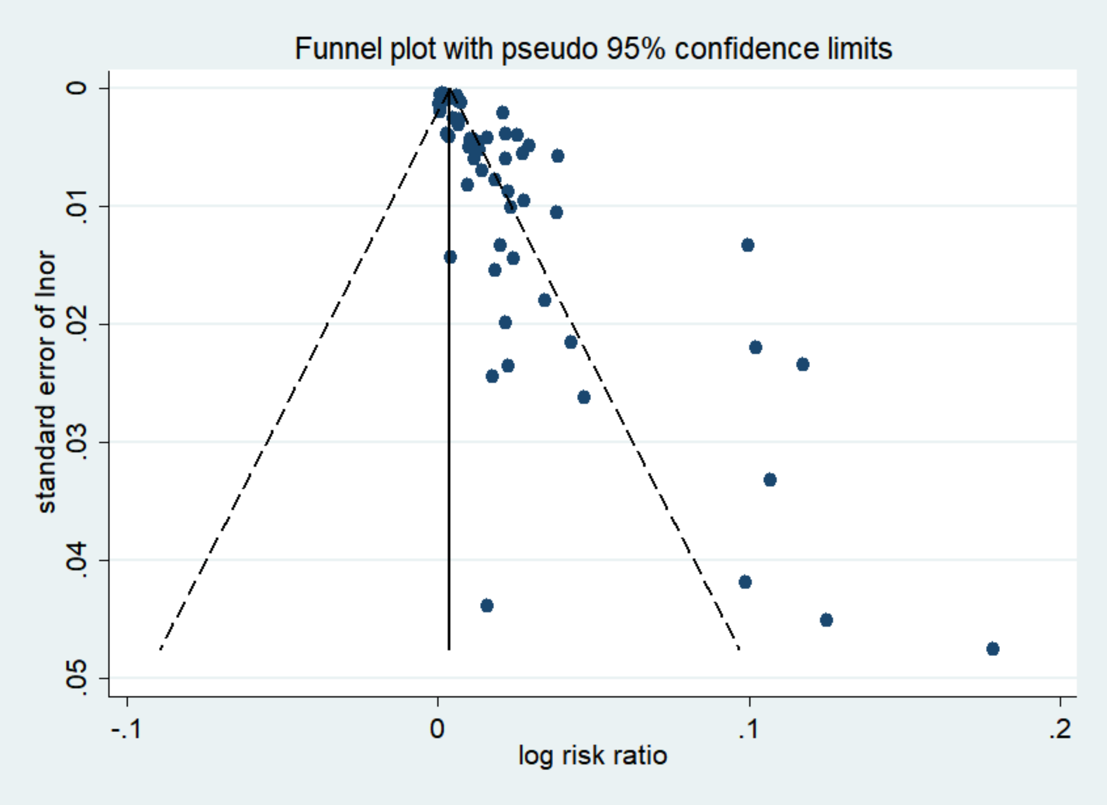

Figure S9: Funnel plots to explore publication bias for studies investigating the association between cold spells and cardiovascular mortality.

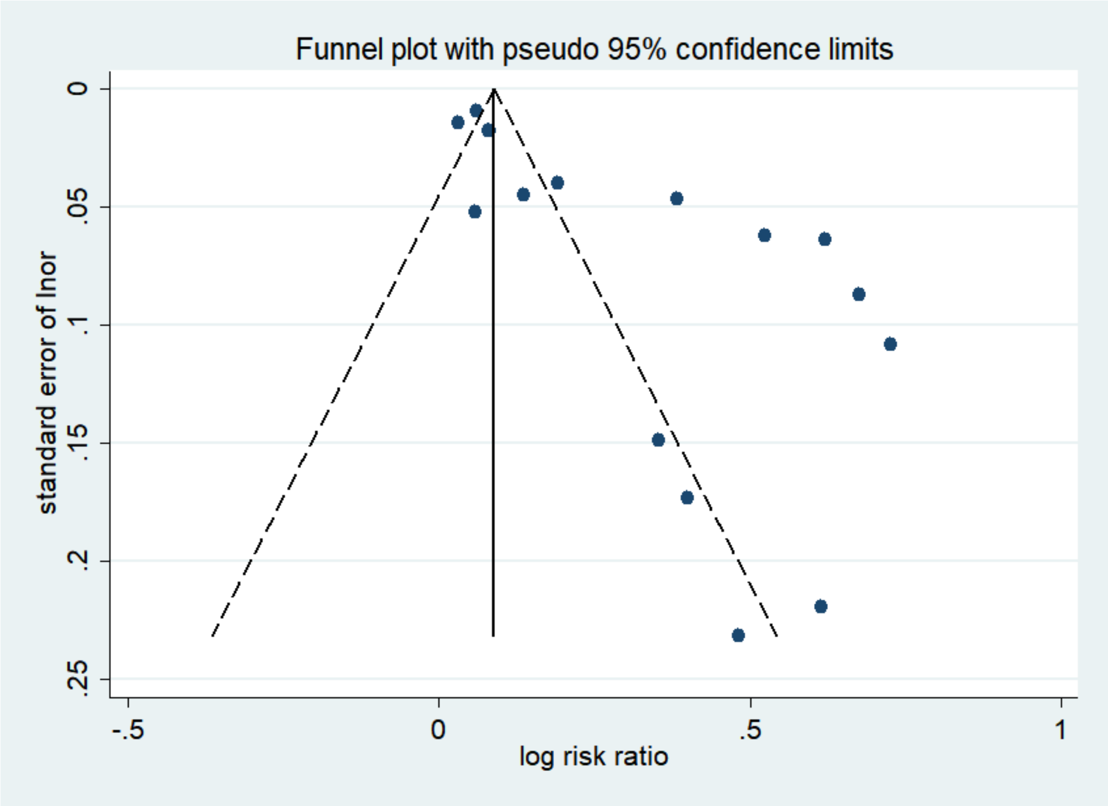

Figure S10: Funnel plots to explore publication bias for studies investigating the association between cold spells and cardiovascular morbidity.

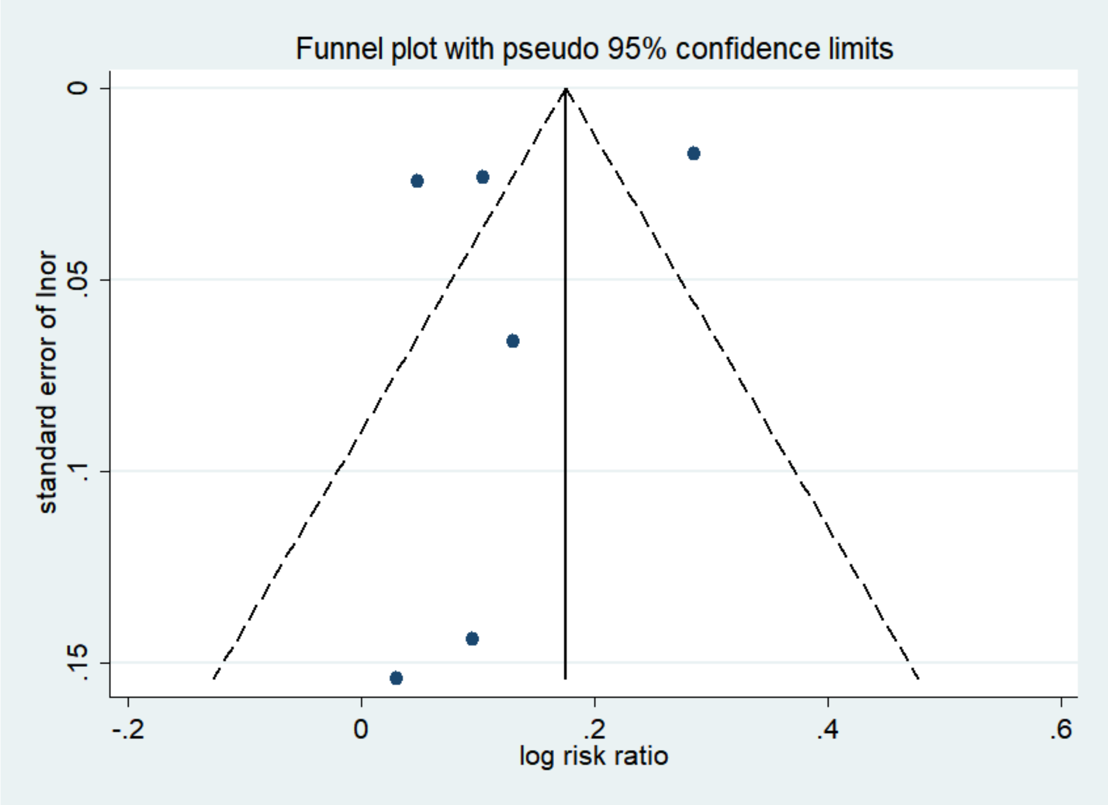

Figure S11: Trim and fill analysis of the studies investigating the association between low temperature and cardiovascular mortality.

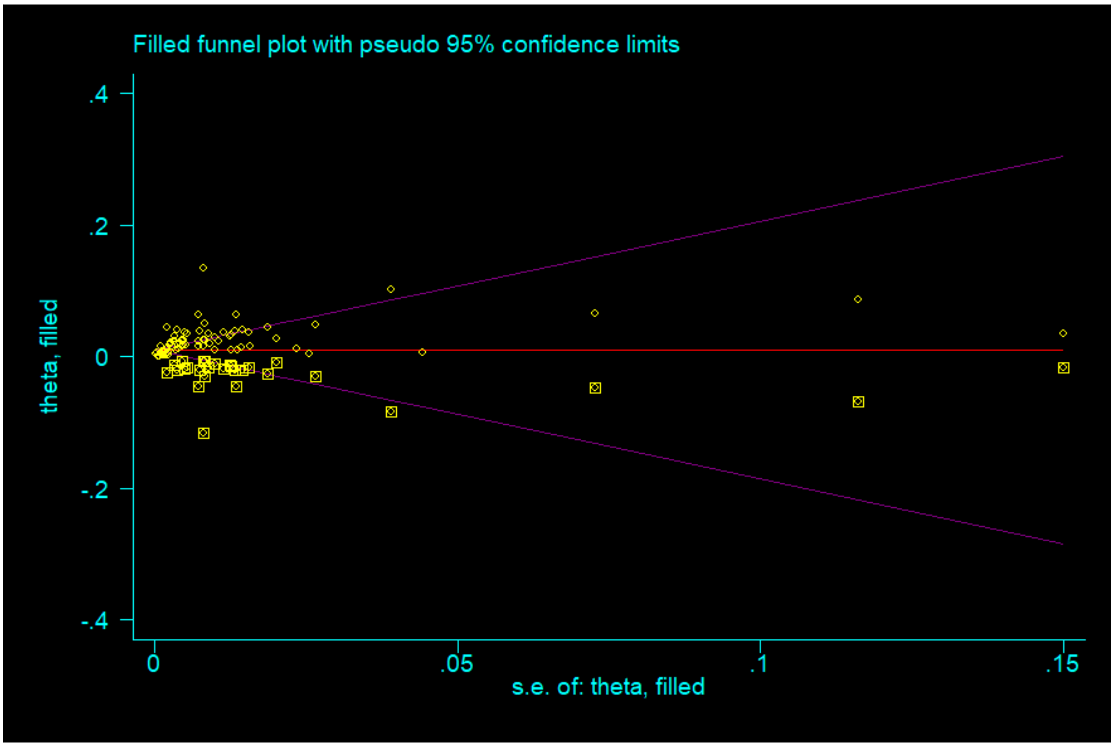

Figure S12: Trim and fill analysis of the studies investigating the association between low temperature and cardiovascular morbidity.

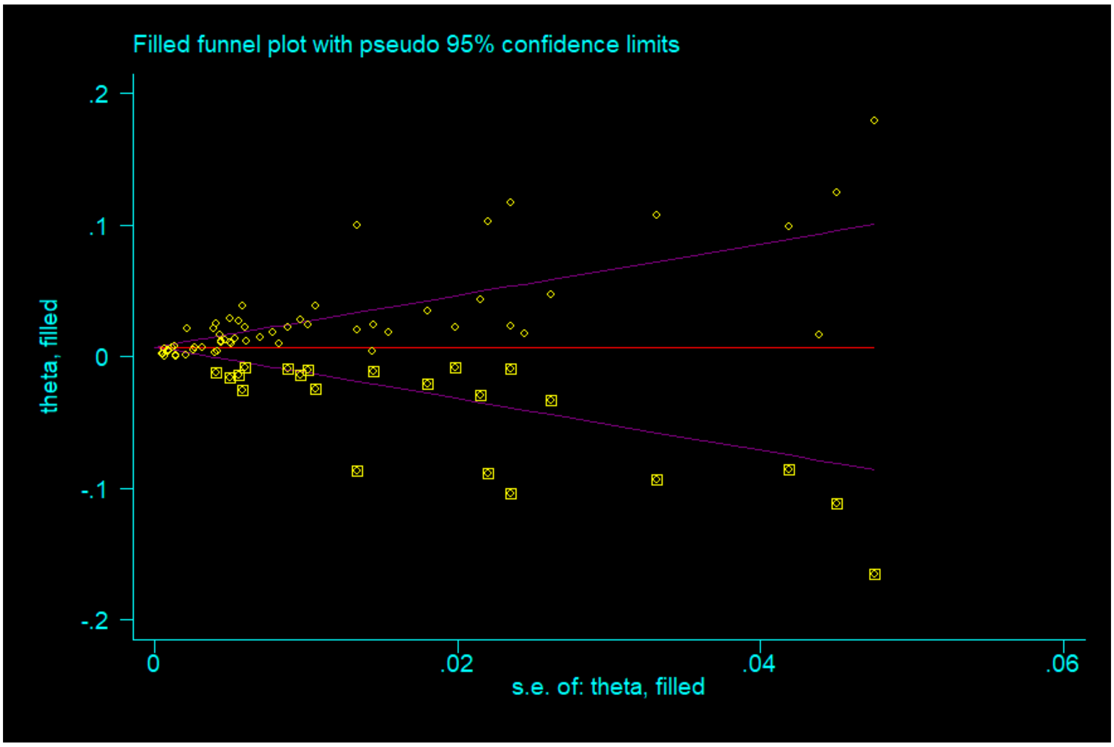

Figure S13: Trim and fill analysis of the studies investigating the association between cold spells and cardiovascular mortality.

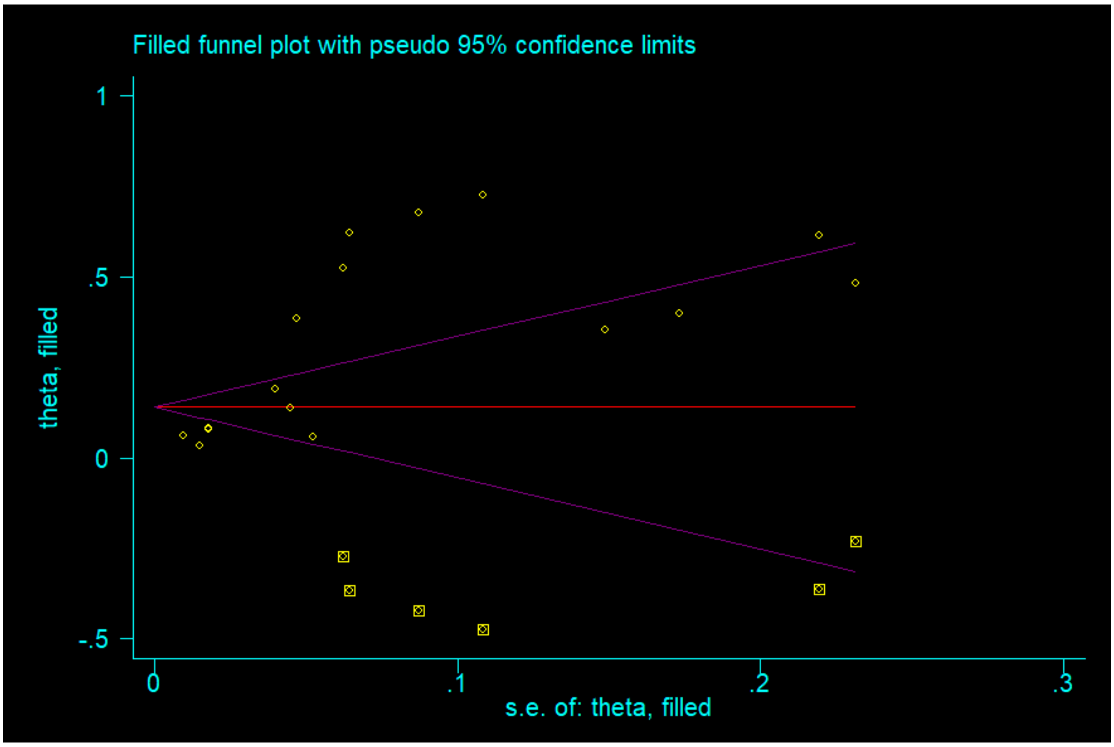

Figure S14: Trim and fill analysis of the studies investigating the association between cold spells and cardiovascular morbidity.

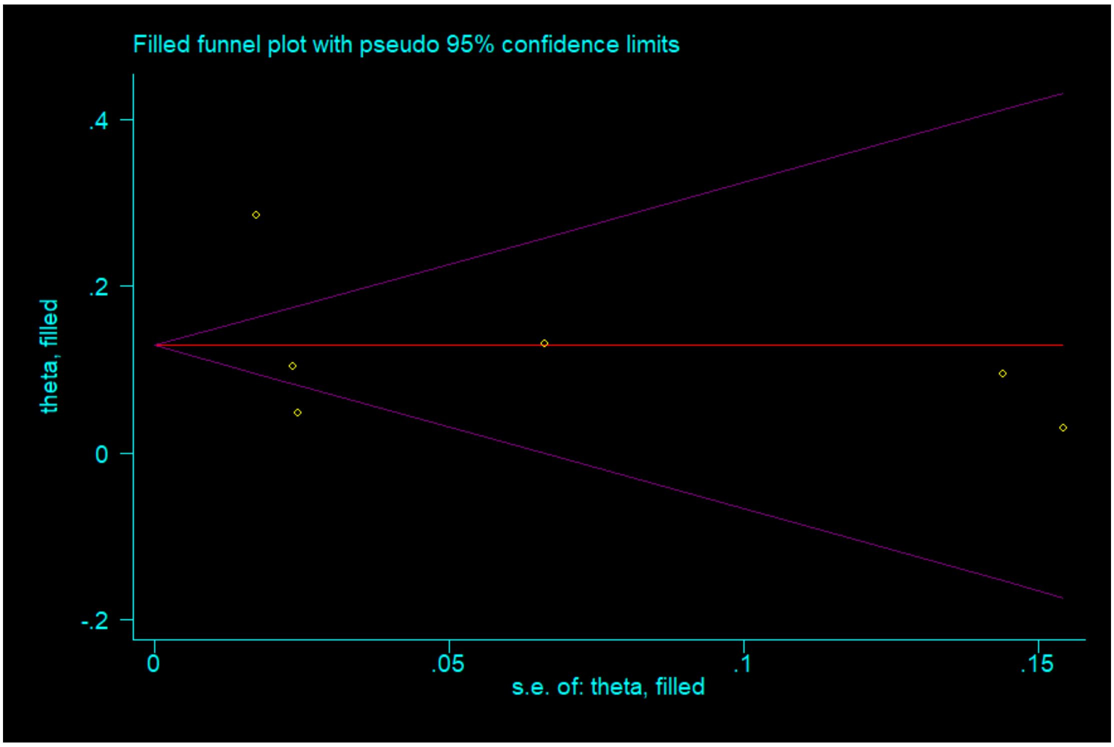

Table S9 Definition of cold spell across studies

**Authors**	**Study Period**	**Cold Spell Definition**

Ma et al.	1972-2015	2d, meanT< 5nd
Rocklov et al.	1990-2002	2d, meanT<2nd
Wu et al.	1994-2003	24h, minT< 10℃
Kysely et al.	1994-2006	10d, MaxT< 3.5℃
Madrigano et al.	1995-2003	3d, mimT< 5th
Chen et al.	1997-2003	24h, minT< 10℃
Ryti et al.	1998-2011	3d, mimT< 5th
Ryti et al.	1998-2011	3d, mimT< 5th
Sartini et al.	1998-2012	3D, mimT< 10th
Revich B. & Shaposhnikov D.	2000-2006	9d, meanT<3th OR 6d, meanT <1st
Vaičiulis ET AL.	2000-2015	2d, meanT<10th
Ma et al.	2001-2009	7d, MaxT< 3rd
Ma et al.	2005-2008	7d, MaxT< 3rd
Zhou et al.	2006-2010	3d, mimT< 5th
Ponjoan et al.	2006-2013	9d, meanT<5th
Moraes et al.	2006-2015	4d, minT< 3nd
Chen et al.	2007-2013	2d, meanT< 3nd
Han et al.	2011-2014	3d, mimT< 5th
Gao et al.	2013-2015	3d, mimT< 5th

Reference

1. Page MJ, McKenzie JE, Bossuyt PM, Boutron I, Hoffmann TC, Mulrow CD, et al. The PRISMA 2020 statement: an updated guideline for reporting systematic reviews. Rev Esp Cardiol (Engl Ed) 2021; 74:790-799.

2. Chen R, Yin P, Wang L, Liu C, Niu Y, Wang W, et al. Association between ambient temperature and mortality risk and burden: time series study in 272 main Chinese cities. Bmj 2018; 363:k4306.

3. Ma C, Yang J, Nak
ayama SF, Iwai-Shimada M, Jung CR, Sun XL, et al. Cold Spells and Cause-Specific Mortality in 47 Japanese Prefectures: A Systematic Evaluation. Environ Health Perspect 2021; 129:67001.

4. Guo Y, Li S, Zhang Y, Armstrong B, Jaakkola JJ, Tong S, et al. Extremely cold and hot temperatures increase the risk of ischaemic heart disease mortality: epidemiological evidence from China. Heart 2013; 99:195-203.

5. Ren C, Williams GM, Tong S. Does particulate matter modify the association between temperature and cardiorespiratory diseases? Environ Health Perspect 2006; 114:1690-1696.

6. Bai L, Cirendunzhu, Woodward A, Dawa, Xiraoruodeng, Liu Q. Temperature and mortality on the roof of the world: a time-series analysis in three Tibetan counties, China. Sci Total Environ 2014; 485-486:41-48.

7. Ha J, Kim H. Changes in the association between summer temperature and mortality in Seoul, South Korea. Int J Biometeorol 2013; 57:535-544

8. Ferreira LCM, Nogueira MC, Pereira RVB, de Farias WCM, Rodrigues MMS, Teixeira MTB, et al. Ambient temperature and mortality due to acute myocardial infarction in Brazil: an ecological study of time-series analyses. Sci Rep 2019; 9:13790.

9. Analitis A, De' Donato F, Scortichini M, Lanki T, Basagana X, Ballester F, et al. Synergistic Effects of

Ambient Temperature and Air Pollution on Health in Europe: Results from the PHASE Project. Int J Environ Res Public Health 2018; 15.
10. Hashizume M, Wagatsuma Y, Hayashi T, Saha SK, Streatfield K, Yunus M. The effect of temperature on mortality in rural Bangladesh--a population-based time-series study. Int J Epidemiol 2009; 38:1689-1697.
11. Huang J, Wang J, Yu W. The lag effects and vulnerabilities of temperature effects on cardiovascular disease mortality in a subtropical climate zone in China. Int J Environ Res Public Health 2014; 11:3982-3994.
12. Lin YK, Chang CK, Wang YC, Ho TJ. Acute and prolonged adverse effects of temperature on mortality from cardiovascular diseases. PLoS One 2013; 8:e82678.
13. Son JY, Gouveia N, Bravo MA, de Freitas CU, Bell ML. The impact of temperature on mortality in a subtropical city: effects of cold, heat, and heat waves in Sao Paulo, Brazil. Int J Biometeorol 2016; 60:113-121.
14. Xiong J, Lan L, Lian Z, Lin Y. Effect of different temperatures on hospital admissions for cardiovascular and cerebrovascular diseases: A case study. Indoor and Built Environment 2015; 26:69-77.
15. Ikefuti PV, Barrozo LV, Braga ALF. Mean air temperature as a risk factor for stroke mortality in Sao Paulo, Brazil. Int J Biometeorol 2018; 62:1535-1542.
16. Gouveia N, Hajat S, Armstrong B. Socioeconomic differentials in the temperature-mortality relationship in Sao Paulo, Brazil. Int J Epidemiol 2003; 32:390-397.
17. Liu S, Chan EYY, Goggins WB, Huang Z. The Mortality Risk and Socioeconomic Vulnerability Associated with High and Low Temperature in Hong Kong. Int J Environ Res Public Health 2020; 17.
18. Guo Y, Barnett AG, Pan X, Yu W, Tong S. The impact of temperature on mortality in Tianjin, China: a case-crossover design with a distributed lag nonlinear model. Environ Health Perspect 2011; 119:1719-1725.
19. Zhang Y, Li S, Pan X, Tong S, Jaakkola JJ, Gasparrini A, et al. The effects of ambient temperature on cerebrovascular mortality: an epidemiologic study in four climatic zones in China. Environ Health 2014; 13:24.
20. Romani SG, Roye D, Sanchez Santos L, Figueiras A. Impact of Extreme Temperatures on Ambulance Dispatches Due to Cardiovascular Causes in North-West Spain. Int J Environ Res Public Health 2020; 17.
21. Dai J, Chen R, Meng X, Yang C, Zhao Z, Kan H. Ambient air pollution, temperature and out-of-hospital coronary deaths in Shanghai, China. Environ Pollut 2015; 203:116-121.
22. Silveira IH, Oliveira BFA, Cortes TR, Junger WL. The effect of ambient temperature on cardiovascular mortality in 27 Brazilian cities. Sci Total Environ 2019; 691:996-1004.
23. Guo Y, Punnasiri K, Tong S. Effects of temperature on mortality in Chiang Mai city, Thailand: a time series study. Environ Health 2012; 11:36.
24. Seposo XT, Dang TN, Honda Y. Evaluating the Effects of Temperature on Mortality in Manila City (Philippines) from 2006-2010 Using a Distributed Lag Nonlinear Model. Int J Environ Res Public Health 2015; 12:6842-6857.
25. Zhang Y, Li C, Feng R, Zhu Y, Wu K, Tan X, et al. The Short-Term Effect of Ambient Temperature on Mortality in Wuhan, China: A Time-Series Study Using a Distributed Lag Non-Linear Model. Int J Environ Res Public Health 2016; 13.
26. Yang J, Ou CQ, Ding Y, Zhou YX, Chen PY. Daily temperature and mortality: a study of distributed lag

non-linear effect and effect modification in Guangzhou. Environ Health 2012; 11:63.
27. Yu W, Mengersen K, Hu W, Guo Y, Pan X, Tong S. Assessing the relationship between global warming and mortality: lag effects of temperature fluctuations by age and mortality categories. Environ Pollut 2011; 159:1789-1793.
28. Yu W, Hu W, Mengersen K, Guo Y, Pan X, Connell D, et al. Time course of temperature effects on cardiovascular mortality in Brisbane, Australia. Heart 2011; 97:1089-1093.
29. Kwon BY, Lee E, Lee S, Heo S, Jo K, Kim J, et al. Vulnerabilities to Temperature Effects on Acute Myocardial Infarction Hospital Admissions in South Korea. Int J Environ Res Public Health 2015; 12:14571-14588.
30. Ma Y, Zhou L, Chen K. Burden of cause-specific mortality attributable to heat and cold: A multicity time-series study in Jiangsu Province, China. Environ Int 2020; 144:105994.
31. Ballester F, Corella D, Perez-Hoyos S, Saez M, Hervas A. Mortality as a function of temperature. A study in Valencia, Spain, 1991-1993. Int J Epidemiol 1997; 26:551-561.
32. Silveira IH, Cortes TR, Oliveira BFA, Junger WL. Temperature and cardiovascular mortality in Rio de Janeiro, Brazil: effect modification by individual-level and neighbourhood-level factors. J Epidemiol Community Health 2021; 75:69-75.
33. Zhai L, Ma X, Wang J, Luan G, Zhang H. Effects of ambient temperature on cardiovascular disease: a time-series analysis of 229288 deaths during 2009-2017 in Qingdao, China. Int J Environ Health Res 2022; 32:181-190.
34. Ma W, Chen R, Kan H. Temperature-related mortality in 17 large Chinese cities: how heat and cold affect mortality in China. Environ Res 2014; 134:127-133.
35. O'Neill MS, Hajat S, Zanobetti A, Ramirez-Aguilar M, Schwartz J. Impact of control for air pollution and respiratory epidemics on the estimated associations of temperature and daily mortality. Int J Biometeorol 2005; 50:121-129.
36. Yi W, Chan AP. Effects of temperature on mortality in Hong Kong: a time series analysis. Int J Biometeorol 2015; 59:927-936.
37. Xing Q, Sun Z, Tao Y, Zhang X, Miao S, Zheng C, et al. Impacts of urbanization on the temperature-cardiovascular mortality relationship in Beijing, China. Environ Res 2020; 191:110234.
38. Sharovsky R, Cesar LA, Ramires JA. Temperature, air pollution, and mortality from myocardial infarction in Sao Paulo, Brazil. Braz J Med Biol Res 2004; 37:1651-1657.
39. Lin YK, Sung FC, Honda Y, Chen YJ, Wang YC. Comparative assessments of mortality from and morbidity of circulatory diseases in association with extreme temperatures. Sci Total Environ 2020; 723:138012.
40. Analitis A, Katsouyanni K, Biggeri A, Baccini M, Forsberg B, Bisanti L, et al. Effects of cold weather on mortality: results from 15 European cities within the PHEWE project. Am J Epidemiol 2008; 168:1397-1408.
41. Denpetkul T, Phosri A. Daily ambient temperature and mortality in Thailand: Estimated effects, attributable risks, and effect modifications by greenness. Sci Total Environ 2021; 791:148373.
42. Chen TH, Li X, Zhao J, Zhang K. Impacts of cold weather on all-cause and cause-specific mortality in Texas, 1990-2011. Environ Pollut 2017; 225:244-251.
43. Antunes L, Silva SP, Marques J, Nunes B, Antunes S. The effect of extreme cold temperatures on the risk of death in the two major Portuguese cities. Int J Biometeorol 2017; 61:127-135.
44. Medina-Ramon M, Schwartz J. Temperature, temperature extremes, and mortality: a study of acclimatisation and effect modification in 50 US cities. Occup Environ Med 2007; 64:827-833.

45. Tian Z, Li S, Zhang J, Jaakkola JJ, Guo Y. Ambient temperature and coronary heart disease mortality in Beijing, China: a time series study. Environ Health 2012; 11:56.
46. Sharafkhani R, Khanjani N, Bakhtiari B, Jahani Y, Entezar Mahdi R. Diurnal temperature range and mortality in Urmia, the Northwest of Iran. J Therm Biol 2017; 69:281-287.
47. Pan WH, Li LA, Tsai MJ. Temperature extremes and mortality from coronary heart disease and cerebral infarction in elderly Chinese. Lancet 1995; 345:353-355.
48. Yang C, Meng X, Chen R, Cai J, Zhao Z, Wan Y, et al. Long-term variations in the association between ambient temperature and daily cardiovascular mortality in Shanghai, China. Sci Total Environ 2015; 538:524-530.
49. Breitner S, Wolf K, Devlin RB, Diaz-Sanchez D, Peters A, Schneider A. Short-term effects of air temperature on mortality and effect modification by air pollution in three cities of Bavaria, Germany: a time-series analysis. Sci Total Environ 2014; 485-486:49-61.
50. Rodrigues M, Santana P, Rocha A. Effects of extreme temperatures on cerebrovascular mortality in Lisbon: a distributed lag non-linear model. Int J Biometeorol 2019; 63:549-559.
51. Chen R, Li T, Cai J, Yan M, Zhao Z, Kan H. Extreme temperatures and out-of-hospital coronary deaths in six large Chinese cities. J Epidemiol Community Health 2014; 68:1119-1124.
52. Chan EY, Goggins WB, Kim JJ, Griffiths SM. A study of intracity variation of temperature-related mortality and socioeconomic status among the Chinese population in Hong Kong. J Epidemiol Community Health 2012; 66:322-327.
53. Rocklov J, Ebi K, Forsberg B. Mortality related to temperature and persistent extreme temperatures: a study of cause-specific and age-stratified mortality. Occup Environ Med 2011; 68:531-536.
54. Fu SH, Gasparrini A, Rodriguez PS, Jha P. Mortality attributable to hot and cold ambient temperatures in India: a nationally representative case-crossover study. PLoS Med 2018; 15:e1002619.
55. Goodman PG, Dockery DW, Clancy L. Cause-specific mortality and the extended effects of particulate pollution and temperature exposure. Environ Health Perspect 2004; 112:179-185.
56. Liu L, Breitner S, Pan X, Franck U, Leitte AM, Wiedensohler A, et al. Associations between air temperature and cardio-respiratory mortality in the urban area of Beijing, China: a time-series analysis. Environ Health 2011; 10:51.
57. Alahmad B, Shakarchi AF, Khraishah H, Alseaidan M, Gasana J, Al-Hemoud A, et al. Extreme temperatures and mortality in Kuwait: Who is vulnerable? Sci Total Environ 2020; 732:139289.
58. Iranpour S, Khodakarim S, Shahsavani A, Khosravi A, Etemad K. Modification of the effect of ambient air temperature on cardiovascular and respiratory mortality by air pollution in Ahvaz, Iran. Epidemiol Health 2020; 42:e2020053.
59. Yin Q, Wang J, Su J, Wei Z. A new method to estimate the temperature-CVD mortality relationship. Environ Sci Pollut Res Int 2019; 26:8895-8901.
60. Hu K, Guo Y, Hochrainer-Stigler S, Liu W, See L, Yang X, et al. Evidence for Urban-Rural Disparity in Temperature-Mortality Relationships in Zhejiang Province, China. Environ Health Perspect 2019; 127:37001.
61. Yang J, Yin P, Zhou M, Ou CQ, Guo Y, Gasparrini A, et al. Cardiovascular mortality risk attributable to ambient temperature in China. Heart 2015; 101:1966-1972.
62. Chen R, Wang C, Meng X, Chen H, Thach TQ, Wong CM, et al. Both low and high temperature may increase the risk of stroke mortality. Neurology 2013; 81:1064-1070.
63. Zhang W, Du G, Xiong L, Liu T, Zheng Z, Yuan Q, et al. Extreme temperatures and cardiovascular

mortality: assessing effect modification by subgroups in Ganzhou, China. Glob Health Action 2021; 14:1965305.
64. Rocklov J, Forsberg B. The effect of temperature on mortality in Stockholm 1998--2003: a study of lag structures and heatwave effects. Scand J Public Health 2008; 36:516-523.
65. Yatim ANM, Latif MT, Sofwan NM, Ahamad F, Khan MF, Mahiyuddin WRW, et al. The association between temperature and cause-specific mortality in the Klang Valley, Malaysia. Environ Sci Pollut Res Int 2021; 28:60209-60220.
66. Xu R, Shi C, Wei J, Lu W, Li Y, Liu T, et al. Cause-specific cardiovascular disease mortality attributable to ambient temperature: A time-stratified case-crossover study in Jiangsu province, China. Ecotoxicol Environ Saf 2022; 236:113498.
67. Schulte F, Roosli M, Ragettli MS. Heat-related cardiovascular morbidity and mortality in Switzerland: a clinical perspective. Swiss Med Wkly 2021; 151:w30013.
68. Lu P, Xia G, Zhao Q, Xu R, Li S, Guo Y. Temporal trends of the association between ambient temperature and hospitalisations for cardiovascular diseases in Queensland, Australia from 1995 to 2016: A time-stratified case-crossover study. PLoS Med 2020; 17:e1003176.
69. Wang X, Li G, Liu L, Westerdahl D, Jin X, Pan X. Effects of Extreme Temperatures on Cause-Specific Cardiovascular Mortality in China. Int J Environ Res Public Health 2015; 12:16136-16156.
70. Polcaro-Pichet S, Kosatsky T, Potter BJ, Bilodeau-Bertrand M, Auger N. Effects of cold temperature and snowfall on stroke mortality: A case-crossover analysis. Environ Int 2019; 126:89-95.
71. von Klot S, Zanobetti A, Schwartz J. Influenza epidemics, seasonality, and the effects of cold weather on cardiac mortality. Environ Health 2012; 11:74.
72. Moghadamnia MT, Ardalan A, Mesdaghinia A, Naddafi K, Yekaninejad MS. The Effects of Apparent Temperature on Cardiovascular Mortality Using a Distributed Lag Nonlinear Model Analysis: 2005 to 2014. Asia Pac J Public Health 2018; 30:361-368.
73. Breitner S, Wolf K, Peters A, Schneider A. Short-term effects of air temperature on cause-specific cardiovascular mortality in Bavaria, Germany. Heart 2014; 100:1272-1280.
74. Chen K, Breitner S, Wolf K, Hampel R, Meisinger C, Heier M, et al. Temporal variations in the triggering of myocardial infarction by air temperature in Augsburg, Germany, 1987-2014. Eur Heart J 2019; 40:1600-1608.
75. Nafstad P, Skrondal A, Bjertness E. Mortality and temperature in Oslo, Norway, 1990-1995. Eur J Epidemiol 2001; 17:621-627.
76. Zhang H, Wang Q, Zhang Y, Yang Y, Zhao Y, Sang J, et al. Modeling the impacts of ambient temperatures on cardiovascular mortality in Yinchuan: evidence from a northwestern city of China. Environ Sci Pollut Res Int 2018; 25:6036-6043.
77. Gholampour R, Darand M, Halabian AH. Impacts of cold and hot temperatures on mortality rate in Isfahan, Iran. J Therm Biol 2019; 86:102453.
78. Tsoutsoubi L, Ioannou LG, Flouris AD. Mortality due to circulatory causes in hot and cold environments in Greece. Scand Cardiovasc J 2021; 55:333-335.
79. Kim H, Heo J, Kim H, Lee JT. Has the impact of temperature on mortality really decreased over time? Sci Total Environ 2015; 512-513:74-81.
80. Anderson BG, Bell ML. Weather-related mortality: how heat, cold, and heat waves affect mortality in the United States. Epidemiology 2009; 20:205-213.
81. Saucy A, Ragettli MS, Vienneau D, de Hoogh K, Tangermann L, Schaffer B, et al. The role of extreme temperature in cause-specific acute cardiovascular mortality in Switzerland: A case-crossover study.

Sci Total Environ 2021; 790:147958.
82. Ebi KL, Exuzides KA, Lau E, Kelsh M, Barnston A. Weather changes associated with hospitalizations for cardiovascular diseases and stroke in California, 1983-1998. Int J Biometeorol 2004; 49:48-58.
83. Rocklov J, Forsberg B, Ebi K, Bellander T. Susceptibility to mortality related to temperature and heat and cold wave duration in the population of Stockholm County, Sweden. Glob Health Action 2014; 7:22737.
84. Wang Q, Gao C, Wang H, Lang L, Yue T, Lin H. Ischemic stroke hospital admission associated with ambient temperature in Jinan, China. PLoS One 2013; 8:e80381.
85. Hajat S, Haines A. Associations of cold temperatures with GP consultations for respiratory and cardiovascular disease amongst the elderly in London. Int J Epidemiol 2002; 31:825-830.
86. Shaposhnikov D, Revich B, Gurfinkel Y, Naumova E. The influence of meteorological and geomagnetic factors on acute myocardial infarction and brain stroke in Moscow, Russia. Int J Biometeorol 2014; 58:799-808.
87. Kovats RS, Hajat S, Wilkinson P. Contrasting patterns of mortality and hospital admissions during hot weather and heat waves in Greater London, UK. Occup Environ Med 2004; 61:893-898.
88. Wu PC, Lin CY, Lung SC, Guo HR, Chou CH, Su HJ. Cardiovascular mortality during heat and cold events: determinants of regional vulnerability in Taiwan. Occup Environ Med 2011; 68:525-530.
89. Kysely J, Pokorna L, Kyncl J, Kriz B. Excess cardiovascular mortality associated with cold spells in the Czech Republic. BMC Public Health 2009; 9:19.
90. Madrigano J, Mittleman MA, Baccarelli A, Goldberg R, Melly S, von Klot S, et al. Temperature, myocardial infarction, and mortality: effect modification by individual- and area-level characteristics. Epidemiology 2013; 24:439-446.
91. Lu P, Zhao Q, Xia G, Xu R, Hanna L, Jiang J, et al. Temporal trends of the association between ambient temperature and cardiovascular mortality: a 17-year case-crossover study. Environmental Research Letters 2021; 16:045004.
92. Bai L, Li Q, Wang J, Lavigne E, Gasparrini A, Copes R, et al. Increased coronary heart disease and stroke hospitalisations from ambient temperatures in Ontario. Heart 2018; 104:673-679.
93. Chen VY, Wu PC, Yang TC, Su HJ. Examining non-stationary effects of social determinants on cardiovascular mortality after cold surges in Taiwan. Sci Total Environ 2010; 408:2042-2049.
94. Martinez-Solanas E, Basagana X. Temporal changes in the effects of ambient temperatures on hospital admissions in Spain. PLoS One 2019; 14:e0218262.
95. Mohammad KN, Chan EYY, Wong MCS, Goggins WB, Chong KC. Ambient temperature, seasonal influenza and risk of cardiovascular disease in a subtropical area in Southern China. Environ Res 2020; 186:109546.
96. Ryti NRI, Makikyro EMS, Antikainen H, Hookana E, Junttila MJ, Ikaheimo TM, et al. Risk of sudden cardiac death in relation to season-specific cold spells: a case-crossover study in Finland. BMJ Open 2017; 7:e017398.
97. Ryti NRI, Junttila MJ, Antikainen H, Kortelainen ML, Huikuri HV, Jaakkola JJK. Coronary stenosis as a modifier of the effect of cold spells on the risk of sudden cardiac death: a case-crossover study in Finland. BMJ Open 2018; 8:e020865.
98. Sartini C, Barry SJE, Wannamethee SG, Whincup PH, Lennon L, Ford I, et al. Effect of cold spells and their modifiers on cardiovascular disease events: Evidence from two prospective studies. Int J Cardiol 2016; 218:275-283.
99. Wichmann J, Ketzel M, Ellermann T, Loft S. Apparent temperature and acute myocardial infarction

hospital admissions in Copenhagen, Denmark: a case-crossover study. Environ Health 2012; 11:19.
100. Wang YC, Lin YK. Association between temperature and emergency room visits for cardiorespiratory diseases, metabolic syndrome-related diseases, and accidents in metropolitan Taipei. PLoS One 2014; 9:e99599.
101. Liang WM, Liu WP, Chou SY, Kuo HW. Ambient temperature and emergency room admissions for acute coronary syndrome in Taiwan. Int J Biometeorol 2008; 52:223-229.
102. Revich B, Shaposhnikov D. Excess mortality during heat waves and cold spells in Moscow, Russia. Occup Environ Med 2008; 65:691-696.
103. Dahlquist M, Raza A, Bero-Bedada G, Hollenberg J, Lind T, Orsini N, et al. Short-term departures from an optimum ambient temperature are associated with increased risk of out-of-hospital cardiac arrest. Int J Hyg Environ Health 2016; 219:389-397.
104. Lin YK, Zafirah Y, Ke MT, Andhikaputra G, Wang YC. The effects of extreme temperatures on emergency room visits-a population-based analysis by age, sex, and comorbidity. Int J Biometeorol 2021; 65:2087-2098.
105. Vaiciulis V, Jaakkola JJK, Radisauskas R, Tamosiunas A, Luksiene D, Ryti NRI. Association between winter cold spells and acute myocardial infarction in Lithuania 2000-2015. Sci Rep 2021; 11:17062.
106. Ma W, Yang C, Chu C, Li T, Tan J, Kan H. The impact of the 2008 cold spell on mortality in Shanghai, China. Int J Biometeorol 2013; 57:179-184.
107. Wichmann J, Andersen Z, Ketzel M, Ellermann T, Loft S. Apparent temperature and cause-specific emergency hospital admissions in Greater Copenhagen, Denmark. PLoS One 2011; 6:e22904.
108. Goggins WB, Chan EY. A study of the short-term associations between hospital admissions and mortality from heart failure and meteorological variables in Hong Kong: Weather and heart failure in Hong Kong. Int J Cardiol 2017; 228:537-542.
109. Kim A, Jung J, Hong J, Yoon SJ. Time series analysis of meteorological factors and air pollutants and their association with hospital admissions for acute myocardial infarction in Korea. Int J Cardiol 2021; 322:220-226.
110. Misailidou M, Pitsavos C, Panagiotakos DB, Chrysohoou C, Stefanadis C. Short-term effects of atmospheric temperature and humidity on morbidity from acute coronary syndromes in free of air pollution rural Greece. Eur J Cardiovasc Prev Rehabil 2006; 13:846-848.
111. Vasconcelos J, Freire E, Almendra R, Silva GL, Santana P. The impact of winter cold weather on acute myocardial infarctions in Portugal. Environ Pollut 2013; 183:14-18.
112. Son JY, Bell ML, Lee JT. The impact of heat, cold, and heat waves on hospital admissions in eight cities in Korea. Int J Biometeorol 2014; 58:1893-1903.
113. Ma W, Xu X, Peng L, Kan H. Impact of extreme temperature on hospital admission in Shanghai, China. Sci Total Environ 2011; 409:3634-3637.
114. Cho SK, Sohn J, Cho J, Noh J, Ha KH, Choi YJ, et al. Effect of Socioeconomic Status and Underlying Disease on the Association between Ambient Temperature and Ischemic Stroke. Yonsei Med J 2018; 59:686-692.
115. Yamazaki S, Michikawa T. Association between high and low ambient temperature and out-of-hospital cardiac arrest with cardiac etiology in Japan: a case-crossover study. Environ Health Prev Med 2017; 22:60.
116. Bai L, Cirendunzhu, Woodward A, Dawa, Zhaxisangmu, Chen B, et al. Temperature, hospital admissions and emergency room visits in Lhasa, Tibet: a time-series analysis. Sci Total Environ 2014; 490:838-848.

117. Tian L, Qiu H, Sun S, Lin H. Emergency Cardiovascular Hospitalization Risk Attributable to Cold Temperatures in Hong Kong. Circ Cardiovasc Qual Outcomes 2016; 9:135-142.
118. Xu Z, Tong S, Pan H, Cheng J. Associations of extreme temperatures with hospitalizations and post-discharge deaths for stroke: What is the role of pre-existing hyperlipidemia? Environ Res 2021; 193:110391.
119. Moghadamnia MT, Ardalan A, Mesdaghinia A, Naddafi K, Yekaninejad MS. Association between apparent temperature and acute coronary syndrome admission in Rasht, Iran. Heart Asia 2018; 10:e011068.
120. Onozuka D, Hagihara A. Out-of-hospital cardiac arrest risk attributable to temperature in Japan. Sci Rep 2017; 7:39538.
121. Shin J, Oh J, Kang IS, Ha E, Pyun WB. Effect of Short-Term Exposure to Fine Particulate Matter and Temperature on Acute Myocardial Infarction in Korea. Int J Environ Res Public Health 2021; 18.
122. Cheng J, Bambrick H, Tong S, Su H, Xu Z, Hu W. Winter temperature and myocardial infarction in Brisbane, Australia: Spatial and temporal analyses. Sci Total Environ 2020; 715:136860.
123. Zhou MG, Wang LJ, Liu T, Zhang YH, Lin HL, Luo Y, et al. Health impact of the 2008 cold spell on mortality in subtropical China: the climate and health impact national assessment study (CHINAs). Environ Health 2014; 13:60.
124. Ponjoan A, Blanch J, Alves-Cabratosa L, Marti-Lluch R, Comas-Cufi M, Parramon D, et al. Effects of extreme temperatures on cardiovascular emergency hospitalizations in a Mediterranean region: a self-controlled case series study. Environ Health 2017; 16:32.
125. Lee S, Lee E, Park MS, Kwon BY, Kim H, Jung DH, et al. Short-term effect of temperature on daily emergency visits for acute myocardial infarction with threshold temperatures. PLoS One 2014; 9:e94070.
126. Wang YC, Lin YK, Chen YJ, Hung SC, Zafirah Y, Sung FC. Ambulance Services Associated with Extreme Temperatures and Fine Particles in a Subtropical Island. Sci Rep 2020; 10:2855.
127. Moraes SL, Almendra R, Barrozo LV. Impact of heat waves and cold spells on cause-specific mortality in the city of Sao Paulo, Brazil. Int J Hyg Environ Health 2022; 239:113861.
128. Chen J, Yang J, Zhou M, Yin P, Wang B, Liu J, et al. Cold spell and mortality in 31 Chinese capital cities: Definitions, vulnerability and implications. Environ Int 2019; 128:271-278.
129. Doan TN, Wilson D, Rashford S, Bosley E. Ambient temperatures, heatwaves and out-of-hospital cardiac arrest in Brisbane, Australia. Occup Environ Med 2021.
130. Giang PN, Dung do V, Bao Giang K, Vinhc HV, Rocklov J. The effect of temperature on cardiovascular disease hospital admissions among elderly people in Thai Nguyen Province, Vietnam. Glob Health Action 2014; 7:23649.
131. Niu Y, Chen R, Liu C, Ran P, Chen A, Chen X, et al. The association between ambient temperature and out-of-hospital cardiac arrest in Guangzhou, China. Sci Total Environ 2016; 572:114-118.
132. Thu Dang TA, Wraith D, Bambrick H, Dung N, Truc TT, Tong S, et al. Short - term effects of temperature on hospital admissions for acute myocardial infarction: A comparison between two neighboring climate zones in Vietnam. Environ Res 2019; 175:167-177.
133. Bijelovic S, Dragic N, Bijelovic M, Kovacevic M, Jevtic M, Ninkovic Mrdenovacki O. Impact of climate conditions on hospital admissions for subcategories of cardiovascular diseases. Med Pr 2017; 68:189-197.
134. Sangkharat K, Mahmood MA, Thornes JE, Fisher PA, Pope FD. Impact of extreme temperatures on ambulance dispatches in London, UK. Environ Res 2020; 182:109100.

135. Hensel M, Stuhr M, Geppert D, Kersten JF, Lorenz J, Kerner T. Relationship between ambient temperature and frequency and severity of cardiovascular emergencies: A prospective observational study based on out-of-hospital care data. Int J Cardiol 2017; 228:553-557.
136. Pourshaikhian M, Moghadamnia MT, Yekaninejad MS, Ghanbari A, Rashti AS, Afraz Kamachli S. The effects of meteorological variables on ambulance attendance for cardiovascular diseases in Rasht, Iran. J Therm Biol 2019; 83:150-156.
137. Zhan ZY, Zhong X, Yang J, Ding Z, Xie XX, Zheng ZQ, et al. Effect of apparent temperature on hospitalization from a spectrum of cardiovascular diseases in rural residents in Fujian, China. Environ Pollut 2022; 303:119101.
138. Han J, Liu S, Zhang J, Zhou L, Fang Q, Zhang J, et al. The impact of temperature extremes on mortality: a time-series study in Jinan, China. BMJ Open 2017; 7:e014741.
139. Mohammadi D, Zare Zadeh M, Zare Sakhvidi MJ. Short-term exposure to extreme temperature and risk of hospital admission due to cardiovascular diseases. Int J Environ Health Res 2021; 31:344-354.
140. Zhao Q, Zhao Y, Li S, Zhang Y, Wang Q, Zhang H, et al. Impact of ambient temperature on clinical visits for cardio-respiratory diseases in rural villages in northwest China. Sci Total Environ 2018; 612:379-385.
141. Luo Y, Li H, Huang F, Van Halm-Lutterodt N, Qin X, Wang A, et al. The cold effect of ambient temperature on ischemic and hemorrhagic stroke hospital admissions: A large database study in Beijing, China between years 2013 and 2014-Utilizing a distributed lag non-linear analysis. Environ Pollut 2018; 232:90-96.
142. Guo P, Zheng M, Wang Y, Feng W, Wu J, Deng C, et al. Effects of ambient temperature on stroke hospital admissions: Results from a time-series analysis of 104,432 strokes in Guangzhou, China. Sci Total Environ 2017; 580:307-315.
143. Gao J, Yu F, Xu Z, Duan J, Cheng Q, Bai L, et al. The association between cold spells and admissions of ischemic stroke in Hefei, China: Modified by gender and age. Sci Total Environ 2019; 669:140-147.
144. Lei J, Chen R, Yin P, Meng X, Zhang L, Liu C, et al. Association between Cold Spells and Mortality Risk and Burden: A Nationwide Study in China. Environ Health Perspect 2022; 130:27006.
145. Liu X, Kong D, Fu J, Zhang Y, Liu Y, Zhao Y, et al. Association between extreme temperature and acute myocardial infarction hospital admissions in Beijing, China: 2013-2016. PLoS One 2018; 13:e0204706.
146. Aklilu D, Wang T, Amsalu E, Feng W, Li Z, Li X, et al. Short-term effects of extreme temperatures on cause specific cardiovascular admissions in Beijing, China. Environ Res 2020; 186:109455.
147. Garcia-Lledo A, Rodriguez-Martin S, Tobias A, Alonso-Martin J, Ansede-Cascudo JC, de Abajo FJ. Heat waves, ambient temperature, and risk of myocardial infarction: an ecological study in the Community of Madrid. Rev Esp Cardiol (Engl Ed) 2020; 73:300-306.
148. Guo S, Niu Y, Cheng Y, Chen R, Kan J, Kan H, et al. Association between ambient temperature and daily emergency hospitalizations for acute coronary syndrome in Yancheng, China. Environ Sci Pollut Res Int 2020; 27:3885-3891.
149. Wang ZX, Cheng YB, Wang Y, Wang Y, Zhang XH, Song HJ, et al. Burden of Outpatient Visits Attributable to Ambient Temperature in Qingdao, China. Biomed Environ Sci 2021; 34:395-399.
150. Wang Q, He Y, Hajat S, Cheng J, Xu Z, Hu W, et al. Temperature-sensitive morbidity indicator: consequence from the increased ambulance dispatches associated with heat and cold exposure. Int J

Biometeorol 2021; 65:1871-1880.
151. Cui L, Geng X, Ding T, Tang J, Xu J, Zhai J. Impact of ambient temperature on hospital admissions for cardiovascular disease in Hefei City, China. Int J Biometeorol 2019; 63:723-734.
152. Mohammad MA, Koul S, Rylance R, Frobert O, Alfredsson J, Sahlen A, et al. Association of Weather With Day-to-Day Incidence of Myocardial Infarction: A SWEDEHEART Nationwide Observational Study. JAMA Cardiol 2018; 3:1081-1089.
153. Li N, Ma J, Liu F, Zhang Y, Ma P, Jin Y, et al. Associations of apparent temperature with acute cardiac events and subtypes of acute coronary syndromes in Beijing, China. Sci Rep 2021; 11:15229.
154. Borghei Y, Moghadamnia MT, Sigaroudi AE, Ghanbari A. Association between climate variables (cold and hot weathers, humidity, atmospheric pressures) with out-of-hospital cardiac arrests in Rasht, Iran. J Therm Biol 2020; 93:102702.
155. Li Y, Ji C, Ju H, Han Y. Impact of ambient temperature and atmospheric evaporation on the incidence of acute deep venous thrombosis in the northeast of China. Int Angiol 2017; 36:243-253.
156. Di Blasi C, Renzi M, Michelozzi P, De' Donato F, Scortichini M, Davoli M, et al. Association between air temperature, air pollution and hospital admissions for pulmonary embolism and venous thrombosis in Italy. Eur J Intern Med 2022; 96:74-80.
157. Chen J, Gao Y, Jiang Y, Li H, Lv M, Duan W, et al. Low ambient temperature and temperature drop between neighbouring days and acute aortic dissection: a case-crossover study. Eur Heart J 2022; 43:228-235.
158. Yu X, Xia L, Xiao J, Zheng J, Xu N, Feng X, et al. Association of Daily Mean Temperature and Temperature Variability With Onset Risks of Acute Aortic Dissection. J Am Heart Assoc 2021; 10:e020190.
159. Zhang Q, Peng L, Hu J, Li H, Jiang Y, Fang W, et al. Low temperature and temperature decline increase acute aortic dissection risk and burden: A nationwide case crossover analysis at hourly level among 40,270 patients. Lancet Reg Health West Pac 2022; 28:100562.
160. Ho HC, Wong MS. Urban environmental influences on the temperature-mortality relationship associated mental disorders and cardiorespiratory diseases during normal summer days in a subtropical city. Environ Sci Pollut Res Int 2019; 26:24272-24285.
161. Viswanathan M, Ansari MT, Berkman ND, Chang S, Hartling L, McPheeters M, et al. Assessing the Risk of Bias of Individual Studies in Systematic Reviews of Health Care Interventions. Methods Guide for Effectiveness and Comparative Effectiveness Reviews. Rockville (MD)2008.
162. Viswanathan M, Berkman ND, Dryden DM, Hartling L. Assessing Risk of Bias and Confounding in Observational Studies of Interventions or Exposures: Further Development of the RTI Item Bank. Rockville (MD)2013.
163. Johnson PI, Sutton P, Atchley DS, Koustas E, Lam J, Sen S, et al. The Navigation Guide - evidence-based medicine meets environmental health: systematic review of human evidence for PFOA effects on fetal growth. Environ Health Perspect 2014; 122:1028-1039.
